# Epitope length variants balance protective immune responses and viral escape in HIV-1 infection

**DOI:** 10.1016/j.celrep.2022.110449

**Published:** 2022-03-01

**Authors:** Phillip Pymm, Stefan Tenzer, Edmund Wee, Mirjana Weimershaus, Anne Burgevin, Simon Kollnberger, Jan Gerstoft, Tracy M. Josephs, Kristin Ladell, James E. McLaren, Victor Appay, David A. Price, Lars Fugger, John I. Bell, Hansjörg Schild, Peter van Endert, Maria Harkiolaki, Astrid K.N. Iversen

**Affiliations:** 1 Nuffield Department of Clinical Neurosciences, Division of Clinical Neurology, Weatherall Institute of Molecular Medicine, John Radcliffe Hospital, University of Oxford, Headley Way, Oxford OX3 9DS, UK; 2 Walter and Eliza Hall Institute of Medical Research, University of Melbourne, 1G Royalparade, Parkville, VIC 3052, Australia; 3 Institute of Immunology, University Medical Center of the Johannes-Gutenberg University of Mainz, Langenbeckstrasse 1, 55131 Mainz, Germany; 4 Institut National de la Santé et de la Recherche Médicale, Unité 1151, Université Paris Descartes, Sorbonne Paris Cité, Hôpital Necker, 149 Rue de Severs, 75015 Paris, France; 5 Centre National de la Recherche Scientifique, UMR8253, Université Paris Descartes, Sorbonne Paris Cité, Hôpital Necker, 149 Rue de Severs, 75015 Paris, France; 6 Division of Infection and Immunity, Cardiff University School of Medicine, University Hospital of Wales, Heath Park, CF14 4XN Cardiff, UK; 7 Department of Infectious Diseases, Rigshospitalet, The National University Hospital, Blegdamsvej 9, 2100 Copenhagen, Denmark; 8 Drug Discovery Biology, Monash Institute of Pharmaceutical Sciences, 381 Royal Parade, Parkville, VIC 3052, Australia; 9 Institut National de la Santé et de la Recherche Médicale, Unité 1135, Centre d’Immunologie et des Maladies Infectieuses, Sorbonne Université, Boulevard de l’Hopital, 75013 Paris, France; 10 International Research Center of Medical Sciences, Kumamoto University, 2-2-1 Honjo, Chuo-ku, Kumamoto City 860-0811, Japan; 11 Systems Immunity Research Institute, Cardiff University School of Medicine, University Hospital of Wales, Tenovus Building, CF14 4XN Cardiff, UK; 12 Medical Research Council Human Immunology Unit, Weatherall Institute of Molecular Medicine, University of Oxford, John Radcliffe Hospital, OX3 9DS Oxford, UK; 13 Office of the Regius Professor of Medicine, The Richard Doll Building, University of Oxford, Old Road Campus, OX3 7LF Oxford, UK; 14 Structural Biology Group, Wellcome Trust Centre for Human Genetics, University of Oxford, Old Road Campus, OX3 7LF Oxford, UK; 15 Diamond Light Source, Harwell Science and Innovation Campus, Fermi Avenue, OX11 0DE Didcot, UK

**Keywords:** HLA-B^∗^27:05, HIV-1, cytotoxic T lymphocytes, natural killer cells, KIR3DL1, viral escape, differential antigen processing, peptide competition, immune-response inhibition, crystal structures

## Abstract

Cytotoxic T lymphocyte (CTL) and natural killer (NK) cell responses to a single optimal 10-mer epitope (KK10) in the human immunodeficiency virus type-1 (HIV-1) protein p24Gag are associated with enhanced immune control in patients expressing human leukocyte antigen (HLA)-B^∗^27:05. We find that proteasomal activity generates multiple length variants of KK10 (4–14 amino acids), which bind TAP and HLA-B^∗^27:05. However, only epitope forms ≥8 amino acids evoke peptide length-specific and cross-reactive CTL responses. Structural analyses reveal that all epitope forms bind HLA-B^∗^27:05 via a conserved N-terminal motif, and competition experiments show that the truncated epitope forms outcompete immunogenic epitope forms for binding to HLA-B^∗^27:05. Common viral escape mutations abolish (L136M) or impair (R132K) production of KK10 and longer epitope forms. Peptide length influences how well the inhibitory NK cell receptor KIR3DL1 binds HLA-B^∗^27:05 peptide complexes and how intraepitope mutations affect this interaction. These results identify a viral escape mechanism from CTL and NK responses based on differential antigen processing and peptide competition.

## Highlights

• Proteasomal activity creates many length variants of the protective KK10 HIV-epitope
• All KK10 length variants bind HLA-B^∗^27:05 through a conserved N-terminal motif
• Short KK10 length variants outcompete immunogenic, longer variants for HLA binding
• Epitope variant competition balances protective immune responses and viral escape

## Graphical abstract

**Figure:**
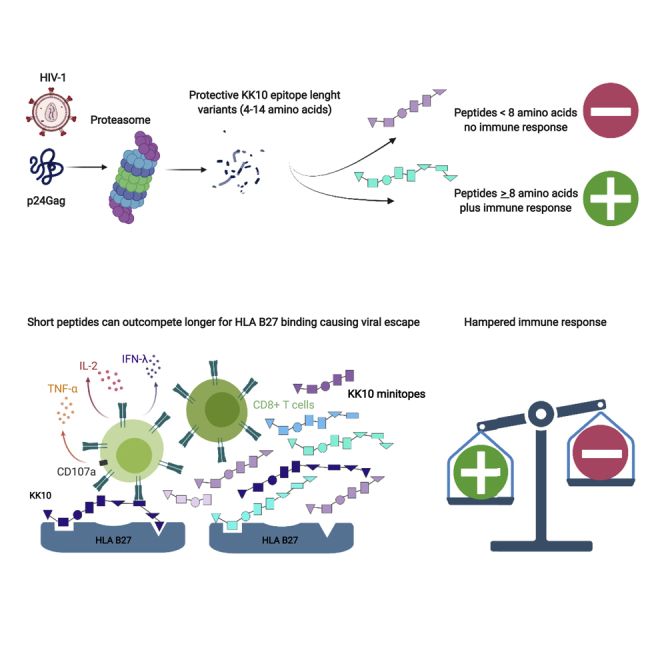

## Teaser

Pymm et al. investigate how HIV-1 adapts to intracellular epitope processing preferences to escape cellular immune responses. Using the processing of the HLA-B^∗^27:05-restricted epitope KK10 as a model, they find that processing generates several epitope length variants and demonstrate that peptide competition can dampen cellular immune responses in the context of HLA-B^∗^27:05.

## Introduction

Human leukocyte antigen (HLA)-B^∗^27:05 and HLA-B^∗^57:01/03 show robust protective effects in human immunodeficiency virus type-1 (HIV-1) infection (Goulder and Walker, 2012). The positive clinical impact of HLA-B^∗^27:05 is linked to CD8^+^ cytotoxic T lymphocyte (CTL) responses directed against a single HIV-1 epitope in the viral capsid protein p24 Gag (^131^KRWIILGLNK^140^) (Feeney et al., 2004; Goulder et al., 1997; Goulder and Watkins, 2008; Nixon et al., 1988). This optimal 10-mer epitope, termed KK10, was defined *in vitro* using progressively truncated peptides to elicit functional responses among peripheral blood mononuclear cells (PBMCs) isolated from HLA-B^∗^27:05^+^ patients (Brander and Walker, 1996; Gnann et al., 1987). Weaker clinical benefits have been associated with the activity of inhibitory natural killer (NK) cells in HLA-B^∗^27:05^+^ patients (Martin and Carrington, 2013; Martin et al., 2007). However, it remains unclear precisely how cellular immune responses directed against a single HIV-1 epitope can delay the onset of acquired immune deficiency syndrome (AIDS) (Goulder and Walker, 2012; Goulder and Watkins, 2008).

Several attributes of KK10-specific CTL responses have been associated with effective immune control of viral replication and slow disease progression, including enhanced antigen sensitivity and polyfunctionality, which in turn have been linked to increased clonal turnover and the expression of particular T cell receptors (TCRs) (Almeida et al., 2007; Chen et al., 2012; Wilson et al., 1998). Other factors may also determine the functional properties of KK10-specific CTLs (Joglekar et al., 2018). However, these studies were focused on the recognition of the optimal KK10 epitope, which is produced in small amounts by human constitutive proteasomes or immunoproteasomes during *in vitro* digestion of longer peptides from p24 Gag (Steers et al., 2011; Tenzer et al., 2009, 2014). In contrast, a range of truncated peptides, known as KK10 minitopes (Tenzer et al., 2009), and C-terminally-extended KK10 epitope forms are produced in abundance (Steers et al., 2011; Tenzer et al., 2009, 2014). Some of these KK10 epitope forms were tested previously in functional assays and failed to elicit functionally superior CTL responses (Tenzer et al., 2009). A structure of the HLA-B^∗^27:05-KK10 complex revealed N-terminal binding of the residue at P2 (R132, or R2_peptide_) in the B pocket, hydrophobic interactions in the E pocket, and C-terminal binding of K10_peptide_ in the F pocket (Stewart-Jones et al., 2005a). However, it remains unclear whether and to what extent truncated and C-terminally-extended KK10 epitope forms can bind HLA-B^∗^27:05.

CTL-induced viral escape mutations can impose fitness costs that suppress HIV-1 replication (Gao et al., 2005; Kiepiela et al., 2007; Migueles and Connors, 2015; Pereyra et al., 2014). In the highly conserved p24 Gag region containing KK10, escape typically occurs via the sequential accumulation of two intraepitope mutations, L136M and R132K. The L136M substitution is thought to impair dendritic-cell-mediated antigen presentation by enhancing the interaction between HLA-B^∗^27:05-KK10 and immunoglobulin-like transcript-4 (ILT-4) (Lichterfeld et al., 2007). This mutation also facilitates viral escape from initially mobilized clonotypes expressing public TCRs (Iglesias et al., 2011; Ladell et al., 2013). The R132K substitution abrogates peptide binding to HLA-B^∗^27:05 (Goulder et al., 1997, 2001; Phillips et al., 1991). However, this mutation incurs a substantial fitness cost, which is partly counteracted by compensatory mutations elsewhere in p24 Gag (Ammaranond et al., 2005; Feeney et al., 2004; Goulder et al., 1997; Schneidewind et al., 2007, 2008, 2009). Epitopes restricted by other HLA class I allotypes overlap the KK10 region but are not associated with enhanced immune control, possibly because cytosolic nardilysin (NRDc) cleavage upstream of the ^131^KR^132^ motif destroys a subset of naturally generated peptides (Kessler et al., 2011).

Here, we investigated the impact of viral escape mutations in p24 Gag on antigen processing and transport and performed structural and functional analyses of naturally produced KK10 epitope forms. Our findings revealed a mechanism of viral escape from CTL and NK responses based on antigen processing of different length variants of KK10 and peptide competition for binding to HLA-B^∗^27:05. As HLA-B27:05 confer substantial inherited risks to several autoimmune diseases, e.g., ankylosing spondylitis and acute anterior uveitis, our results indicate that peptide competition in this context might be used as a therapeutic approach to inhibit or silence errant immune responses.

## Results

### CTL escape mutations distort proteasomal processing of KK10

**Figure 1:**
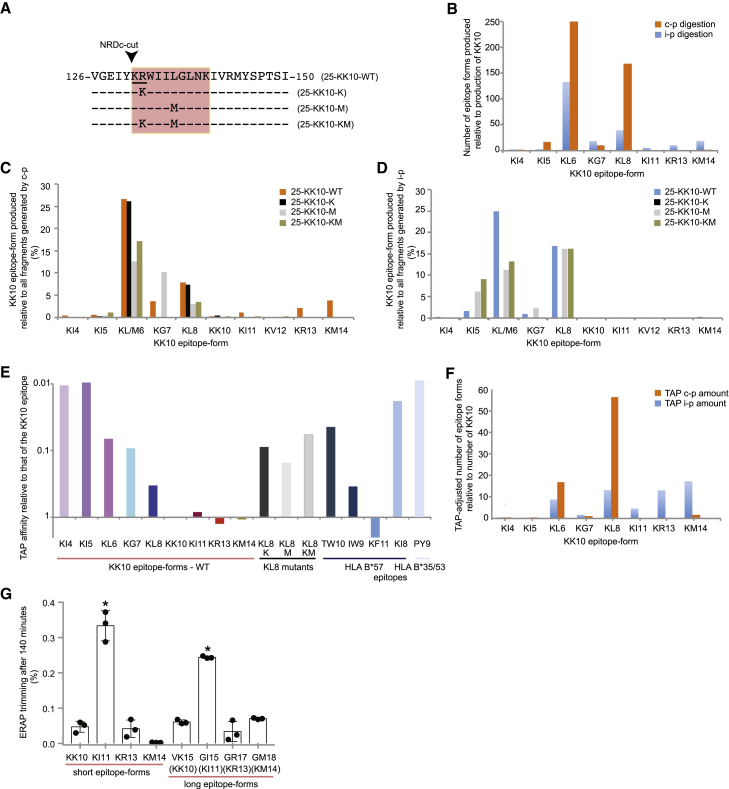
Antigen processing of the p24 Gag region incorporating KK10 (A) Outline of the p24 Gag region (amino acids 126–150). Alignments are shown for common escape mutations in the KK10 epitope (red). NRDc, cytosolic nardilysin. The NRDc recognition motif is underlined (^131^KR^132^). (B) Production of KK10 epitope forms relative to the production of KK10 in proteasomal digests analyzed after 4 h to ensure full representation of all cleavage products (Tenzer et al., 2009, 2014). (C and D) Percentage of KK10 epitope forms made after processing of 25-KK10-WT, 25-KK10-K, 25-KK10-M, or 25-KK10-KM by constitutive proteasomes (C) or immunoproteasomes (D). Proteasomal digests were analyzed after 4 h (Tenzer et al., 2009, 2014). KL/M6 indicates KL6 or KM6 epitope forms. (E) Normalized TAP affinities for KK10 epitope forms relative to KK10. Comparative data are shown for TW10, IW9, KF11, KI8, and PY9, restricted by HLA-B^∗^57:01/03 or HLA-B^∗^35:01 (PY9 only) (HIV Database, 2020). (F) TAP-adjusted production of KK10 epitope forms (details as in B). (G) Percentage reduction in the abundance of KK10 and longer KK10 epitope forms after incubation with ERAP1/2 for 140 min (mean ± SD). See also Table S1. ^∗^p < 0.0001 (one-way ANOVA with Dunnett's test for multiple comparisons). KI11 was trimmed more than GI 15 (p = 0.0005, Mann-Whitney *U* test). c-p, constitutive proteasome; i-p, immunoproteasome. Data are representative of 3–4 independent experiments (B–G).

Common KK10-associated CTL escape mutations (R132K and L136M) typically accumulate late in infection and are associated with sharp increases in viral replication (Feeney et al., 2004). However, the effects of these mutations on epitope processing have not been determined previously. As the HLA-B^∗^27:05 allele is relatively frequent in white people, but rare in Black people (Gonzalez-Galarza et al., 2015), and the HIV-1 subtype B (HIV-B) predominates in white people (HIV Database, 2020) (Kist et al., 2020), we examined the impact of R132K and L136M on proteasomal processing of the KK10 epitope derived from HIV-B. Four 25-mer peptides were synthesized to match the consensus sequence of HIV-B (HIV Database, 2020), with or without one or both of the common escape substitutions: 25-KK10-wild type (WT), 25-KK10-R132 (K), 25-KK10-L136M (M), and 25-KK10-R132K/L136M (KM) (Figure 1A). We performed *in vitro* digestions of these 25-mer peptides using purified constitutive proteasomes and immunoproteasomes (containing subunits β1i, β2i, and β5i) and analyzed the resulting fragments using mass spectrometry (Schmidt et al., 2012; Tenzer et al., 2009, 2014) (Figure 1A). KK10 epitope forms were defined as peptide fragments incorporating the R2 anchor and other segments of KK10. These peptide fragments could be extended by one or several residues at the N- and/or C terminus (Table S1) (Tenzer et al., 2009, 2014).

Constitutive proteasomal and immunoproteasomal digestion of the 25-KK10-WT peptide resulted in the production of KK10 and several epitope forms that were either truncated, including the KK10 minitopes (Tenzer et al., 2009) KRWI (KI4), KRWII (KI5), KRWIIL (KL6), KRWIILG (KG7), and KRWIILGL (KL8), or C-terminally extended, including KRWIILGLNKI (KI11), KRWIILGLNKIVR (KR13), and KRWIILGLNKIVRM (KM14). The production of KK10 minitopes, especially KL6 and KL8, was favored by both proteasomal forms at all time points over the production of KK10 (Figure 1B). In contrast, only KK10 minitopes were generated from the singly substituted 25-mer peptides except for constitutive proteasomal digestion of the 25-KK10-K peptide, which produced small amounts of KK10-K (about 1.4% of all epitope forms) (Figures 1C and 1D; Table S1). Immunoproteosomal digestion of the 25-KK10-K peptide notably generated almost no KK10 epitope forms (<0.3% of all fragments). Constitutive and immunoproteasomal digestion of the doubly substituted 25-mer peptide again favored the production of KK10 minitopes but also generated some C-terminally-extended peptides, in total constituting 0.01% to 2.7% of all epitope forms (Figures 1C and 1D). However, epitope forms containing 132K cannot induce CTL responses because the R132K substitution thwarts KK10 binding to HLA-B^∗^27:05 (Goulder et al., 2001; Phillips et al., 1991).

Proteasomal digestion of the 25-KK10-WT peptide therefore favored the production of KK10 minitopes with suboptimal HLA class I epitope lengths (Paul et al., 2013), and the presence of CTL escape mutations either skewed this preference further (R132K) or resulted in the exclusive production of KK10 minitopes (L136M).

### Efficient binding of KK10 epitope forms to TAP

To induce a CTL response, the transporter associated with antigen processing (TAP) must transport processed KK10 epitope forms into the endoplasmic reticulum (ER). As TAP binding, not translocation, is the rate-limiting step (Gubler et al., 1998), we determined the normalized TAP affinities for all KK10 epitope forms. The results were adjusted to show epitope-form affinity relative to that of KK10 (Figure 1E).

TAP bound all KK10 epitope forms with a low-to-high affinity (Lauvau et al., 1999), which typically increased with epitope length. Even the binding of epitope forms with the lowest affinities (KI4 and KI5) was comparable to that of the immunodominant HLA-B^∗^57:01/B^∗^5703-restricted HIV-1 epitope KI8 (Goulder et al., 2000; Kloverpris et al., 2012) and the immunodominant HLA B^∗^35:01-restricted HIV-1 epitope PY9 (Matthews et al., 2012). The affinities of KL6, KG7, and KL8 exceeded that of the immunodominant HLA-B^∗^57:01/B^∗^5703-restricted HIV-1 epitope TW10 (Goulder and Walker, 2012), and KL8 mutants bound TAP with intermediate affinity (Figure 1E).

Adjusting for TAP interactions changed the predicted relative abundance of epitope forms between the cytosol and the ER, such that KL8 became the dominant minitope, whereas KL6 was the most abundant epitope form after proteasomal digestion (Figures 1B and 1F). However, the overall predicted relative values for shorter and longer KK10 epitope forms in the ER still vastly exceeded that of KK10 (Figure 1F).

### ERAP1/2 trimming is slow and maintains epitope diversity

After TAP-mediated transport into the ER, ER aminopeptidase (ERAP) might trim KK10 and the longer KK10 epitope forms, most likely before binding to HLA-B^∗^27:05 (van Endert, 2011). To study this effect, we performed ERAP1/2 digestions (Tenzer et al., 2014) of N-terminally-extended epitope forms (Table S1) and epitope forms starting with ^131^K, equivalent to peptides that have undergone NRDc digestion (Figures 1A and 1G) (Kessler et al., 2011). We did not test truncated epitope forms because ERAP1 spares most peptides shorter than 8–9 amino acids (Chang et al., 2005; Saric et al., 2002; York et al., 2002). We observed only minimal ERAP1/2 trimming of most epitope forms (Figure 1G), but peptides ending with an I (KI11 and GI15) were trimmed significantly more than other epitope forms, and KI11 was trimmed faster than GI15. The specificity of ERAP1/2 was therefore likely insufficient to exert a marked effect on the relative abundance of KK10 epitope forms.

### HLA-B^∗^27:05 binds KK10 epitope forms

To examine if and how truncated and C-terminally-extended KK10 epitope forms bind HLA-B^∗^27:05, we refolded epitope-form peptides with the HLA-B^∗^27:05 heavy chain and β2 microglobulin (β2m). High-resolution crystal structures were obtained for HLA-B^∗^27:05 in complex with three KK10 minitopes (KL6, KG7, and KL8) and three extended KK10 epitope forms (KI11, KR13, and KM14) (Table S2; Figure S1). However, we were unable to crystallize HLA-B^∗^27:05-KI4 and HLA-B^∗^27:05-KL5, despite the formation of heterotrimeric complexes that incorporated KI4 or KL5, respectively (Figure S2). To examine if ERAP1/2-digested peptides without the N-terminal R2_peptide_ bound to HLA-B^∗^27:05, we set up refolds with the WIILGLNK (WK8) peptide. These refolds yielded no detectable heterotrimers (Figure S2D), suggesting that occupation of the C terminus alone was insufficient to stabilize HLA-B^∗^27:05 (Xiao et al., 2017). As WK8 is not produced in KK10 digestions, we concluded that all naturally produced KK10 epitope forms bound HLA-B^∗^27:05.

### Three N-terminal amino acids are sufficient to stabilize HLA-B^∗^27:05

**Figure 2:**
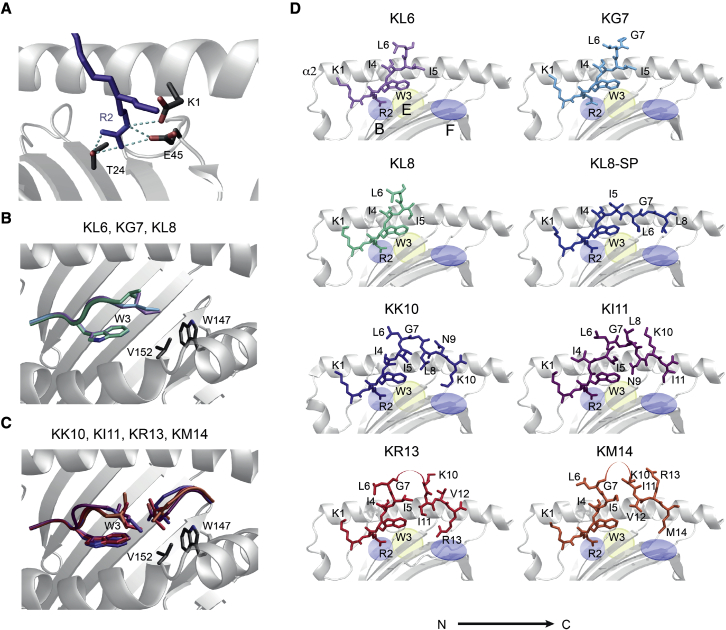
Shared N-terminal structural motif and side views of KK10 epitope forms complexed with HLA-B^∗^27:05 (A) Model of the conserved N-terminal binding region in the HLA-B^∗^27:05 groove. R2_peptide_ was stabilized via the formation of a planar hydrogen bond network between the E45_B27_ Oε_2_ and the R2_epitope_ Nη_2_ and Nε atoms and between the R2_epitope_ Nη_1_ and Nη_2_ atoms and the T24_B27_ Oγ atom. (B and C) Model of W3_peptide_ interactions with W147_B27_ and V152_B27_ in the hydrophobic E pocket. These interactions were similar for truncated (B) and extended (C) KK10 epitope forms. (D) Side views of the peptide-binding groove of HLA-B^∗^27:05 complexed with KK10 epitope forms. The structure of HLA-B^∗^27:05-KK10 was reported in Stewart-Jones et al. (2005a) (PDB: 2BSS). KK10 bound to the B- and F pockets of HLA-B^∗^27:05 via anchors at positions 2 and 10 and formed a central peptide bulge (residues 4 and 7). Unmodeled central peptide residues with poor electron density are shown as a connecting arc. KL8 C-terminal unmodeled peptide residues are not shown. The KL8 structure appeared to have two conformations, with a minority population stretched along the floor of the binding groove (KL8-SP). Peptides are shown against the α2_B27_ helix. The α1_B27_ helix was removed for visual clarity. The B- and F pockets are indicated by light-blue disks and the E pocket by a light-yellow disk.

**Figure 3:**
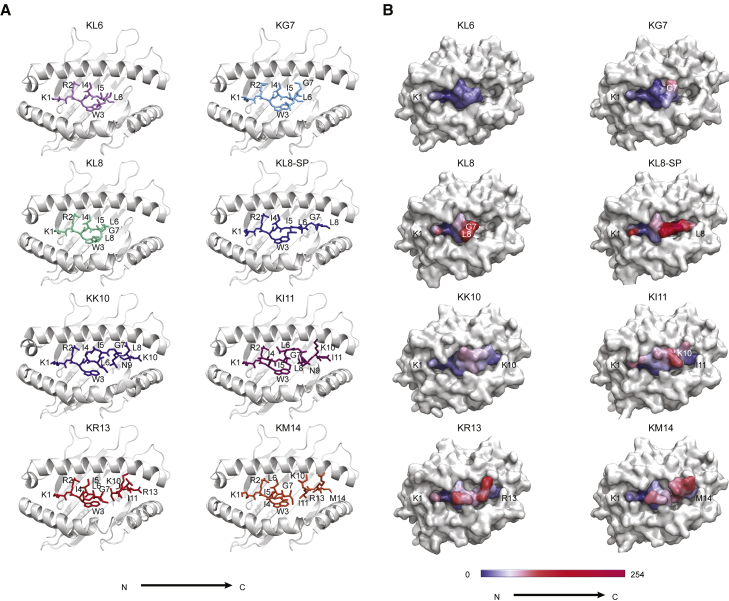
Top views of KK10 epitope forms complexed with HLA-B^∗^27:05 and relative disorder of peptides (A) Top views of the peptide-binding groove of HLA-B^∗^27:05 complexed with KK10 epitope forms. The central peptide bulge in the KR13 and KM14 structures and the residues G7_KL8_ and L8_KL8_ were disordered. (B) The relative local disorder of KK10 epitope-form peptides is derived from the normalized B factors associated with each structure in (A) after normalization of the B factors associated only with HLA-B^∗^27:05 α-chain. Values represent local disorder of the peptide and are colored from blue (no disorder) to increasingly deeper shades of magenta (high disorder).

We compared the seven KK10 epitope-form crystal structures to identify shared and distinct molecular features that might impact T and/or NK cell recognition. In each case, the N-terminal residue bound in the A pocket of HLA-B^∗^27:05 (Figure S3), and the R2_peptide_ anchor docked in the B pocket, forming a network of hydrogen bonds and salt bridges with K1_peptide_, E45_B27_, and T24_B27_ (Figure 2A). The W3_peptide_ stacked against Y159_B27_ and L156_B27_ in the D pocket and interacted with W147_B27_ and V152_B27_ in the hydrophobic E pocket (Figures 2B and 2C). The I4_peptide_ occupied the C pocket and interacted with A69_B27_ and I66_B27_. Collectively, these interactions resulted in a highly conserved structural motif that differed only in the KI11 structure (Figure 2D). In the KI11 structure, the Cα of the I4_peptide_ was pushed upward and N-terminally compared with I4_KKI0_ and did not interact with A69_B27_. The associated normalized B factors were low, suggesting that this region was quite immobile (Figures 3A and 3B). This region also maintained a similar electrostatic surface potential throughout all structures unlike other regions, with the caveat of analytical limitations imposed by the bulging and truncated peptides (Figure S4).

Although residues 1 to 5 were in contact with the binding groove of HLA-B^∗^27:05 in the KL6, KG7, and KL8 structures, residues 6, 7, and/or 8 were positioned above the groove and were solvent exposed (Figure 2D). The normalized B factors were very high for G7_peptide_ (in the KG7 and KL8 structures) and L8_peptide_ (in the KL8 structure) (Figures 3A and 3B), suggesting that these residues were mobile. In addition, the KL8 structure displayed weak electron density along the floor of the binding groove to the C terminus. This electron density pattern suggested the existence of a minority population (approximately 10%) of heterotrimeric complexes in which the peptide was “stretched” along the binding groove and bound at both the N- and C termini. Accordingly, the KL8 peptide might alternate between the extended “stretched-peptide” (KL8-SP) conformation and a more common partially bound conformation (Figures 2D, 3A, and S5). The lack of a C-terminal anchor in the KL6 and KG7 structures was associated with the outward displacement of the α1_B27_ and α2_B27_ helices, which was greatest at the C-terminal end of the α1_B27_ helix (0.92 Å) (Figure S6). A smaller displacement was observed in the KL8 structure, possibly because the KL8-SP conformation helped stabilize the α1_B27_ and α2_B27_ helices.

Collectively, these data revealed that the interaction between HLA-B^∗^27:05 and the conserved, positively charged N-terminal KK10 region was sufficiently strong to stabilize the truncated peptide-HLA-B∗27:05 complexes.

### Differential binding of long KK10 epitope-form peptides to the E- and F pockets

**Figure 4:**
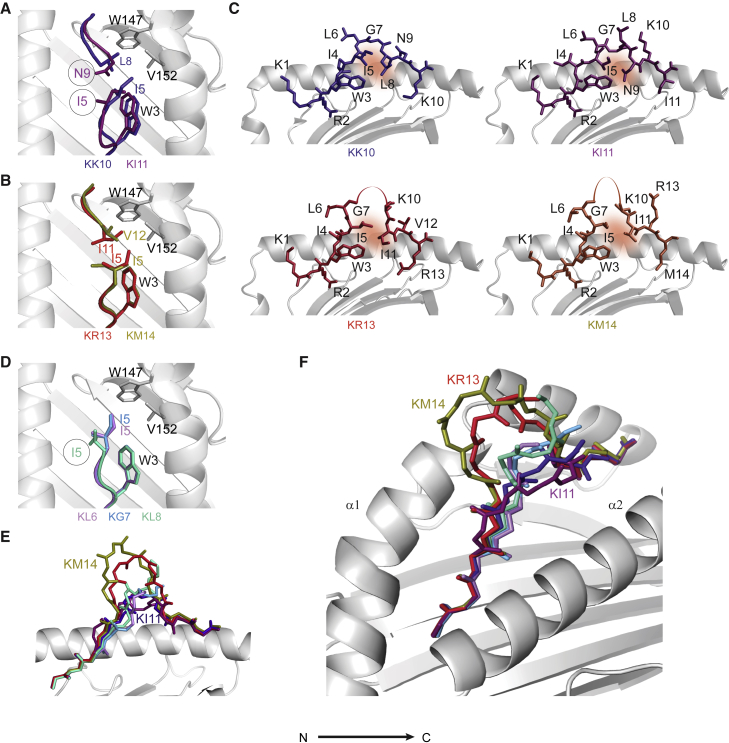
Shared and distinct binding motifs of KK10 epitope forms in the E- and F pockets of HLA-B^∗^27:05 (A and B) Models show the partly distinctive way in which each KK10 epitope form interacted with the E pocket of HLA-B^∗^27:05. Comparisons are shown for KK10 versus KI11 (A) and for KR13 versus KM14 (B). The distance between I5_KI11_ and V152_B27_ was 5 Å. (C) Potentially stabilizing VDW interactions between the N- and C-terminal ends of the central peptide bulge in the KK10, KI11, KR13, and KM14 structures are shaded orange. (D) As in (A) and (B) but comparing KL6, KG7, and KL8. The distance between I5_KL8_ and V152_B27_ was 7.1 Å, which precluded VDW interactions between the amino acid side chains. (E and F) Overlay of all structures with peptide backbones shown (including unmodeled residues). The α2_B27_ helix was removed from the side view (E) but not from the diagonal view (F). The positions of the central bulges in the longer KK10 epitope forms varied in distance from the α2_B27_ helix: KI1 < KK10 < KM14 < KR13.

The hydrophobic patch in the E pocket helped stabilize all epitope forms via interactions with W3_peptide_ (Figures 2B and 2C). This region also formed additional contacts with the longer epitope-form peptides. In the KK10, KR13, and KM14 structures, we observed shared hydrophobic interactions between V152_B27_ and the I5_peptide_ side chain and distinct interactions between V152_B27_ and L8_peptide_ in the KK10 structure (Figure 4A), I11_peptide_ in the KR13 structure, and V12_peptide_ in the KM14 structure (Figure 4B). In these structures, I5_peptide_ and the indicated hydrophobic residues were positioned at the N- and C-terminal ends of the central peptide loop, respectively, and the interaction of their side chains with each other and the E pocket potentially stabilized the central peptide loop (Figure 4C).

The KL8 and KI11 peptide interactions with the E pocket were different and appeared to be suboptimal. In the KL8 structure, the I5_peptide_ side chain was rotated and perpendicular to the base of the groove, which increased the distance between the E pocket and the Cδ1 group to >5 Å (Figure 4D). In the KI11 structure, the Cα of the I4_peptide_ and the Cα of the I5_peptide_ were positioned above and more toward the N-terminal of the corresponding residues in the KK10 structure, which increased solvent exposure (Figures 4A and 4C). The position of L8_peptide_ was exceptional in the KI11 structure, as it formed part of the central loop (Figure 4C). Consequently, the hydrophilic N9_peptide_ side chain was directed toward the E pocket but kinked away, which allowed the δ-oxygen and δ-amino groups to interact with the ordered water molecules between the peptide and the α1_B27_ helix (Figure 4C). This distinct arrangement of residues 4 to 9 resulted in a reduced interaction between I5_peptide_ and N9_peptide_ at the N- and C-terminal ends of the central peptide loop compared with that found in the KK10, KR13, and KM14 structures (Figures 4A and 4B) and a highly flexible K10_peptide_ that protruded by approximately 3 Å (Figures 3A and 4C). The distinct E-pocket conformation and the larger central loop relative to KK10 also caused an exaggerated “zigzag” in the KI11 peptide backbone between the α1_B27_ and α2_B27_ helices, with marked tilting toward the α2_B27_ helix compared with KK10 (Figures 4E and 4F). These features resulted in greater overall flexibility and solvent exposure of the central bulge compared with that of KK10, potentially giving rise to more recognition sites for TCRs.

The F pocket of HLA-B^∗^27:05 binds a broader range of amino acids than other HLA-B^∗^27 subtypes and most other HLA class I molecules (HIV Database. 2020). Hydrophobic residues at the entrance and acidic residues at the base allowed binding of either small hydrophobic side chains (L, I, and M in KL8, KI11, and KM14, respectively) or long basic side chains (K and R in KK10 and KR13, respectively). Although the different side chains interacted with distinct residues in the F pocket and carried different electrostatic charges, the peptide backbone conformations were remarkably similar (Figure 4E), and the B factors suggested that this region was relatively stable (Figure 3B). In the minitope structures, some electron density was present in the F pocket, and we identified an arginine in the KG7 structure that was most likely acquired from the refolding buffer and potentially helped stabilize HLA-B^∗^27:05.

Collectively, these analyses revealed similar N- and C-terminal regions and distinct interactions with the E pocket and variable flexibility and solvent exposure in the central peptide loops of the longer epitope forms and further suggested that KL8 could adopt multiple conformations in the binding groove of HLA-B^∗^27:05.

### Patterns of CTL recognition associate with patterns of viral escape

To examine the immunogenicity of naturally produced KK10 epitope forms, we used *ex vivo* interferon (IFN)-γ enzyme-linked immunospot (ELISpot) assays to quantify peptide-specific responses among PBMCs isolated from ten HLA-B^∗^27:05^+^ patients with chronic HIV-1 infection (Table S3) (Tenzer et al., 2009). Specific IFN-γ release was identified in response to KL8, KK10, KI11, KR13, and/or KM14 but not in response to other minitope peptides. The poor immunogenicity of KI4 and KI5 could be explained by poor or absent HLA-B^∗^27:05 binding because these peptides refolded inefficiently *in vitro* with HLA-B^∗^27:05. Similarly, the poor immunogenicity of KL6 and KG7 could be explained by the lack of C-terminal anchor residues and the associated outward displacement of the α1_B27_ and α2_B27_ helices, which might hinder productive TCR interactions.

**Figure 5:**
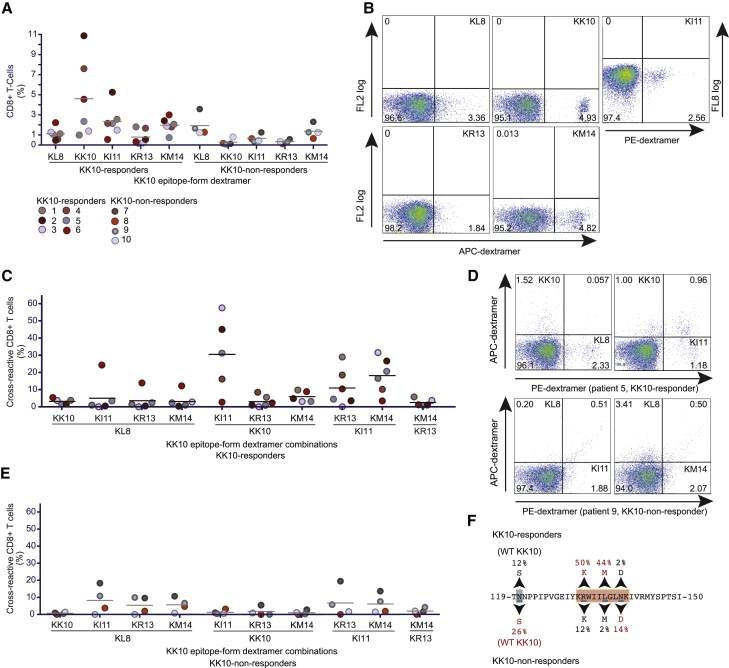
*Ex vivo* HLA-B^∗^27:05-peptide dextramer reactivity and sequence variation in HIV-1 (A) Percentage of HLA-B^∗^27:05-peptide dextramer^+^ CD8^+^ T cells in KK10 responders and KK10 non-responders. (B) Representative flow cytometry plots showing patterns of staining with individual HLA-B^∗^27:05-peptide dextramers with dextramer fluorophore on the x axis (gated on viable CD3^+^ events). Numbers indicate quadrant percentages. (C) Percentage of cross-reactive CD8^+^ T cells in KK10 responders. (D) Representative flow cytometry plots showing cross-reactive staining with HLA-B^∗^27:05-peptide dextramers (gated on viable CD3^+^ CD8^+^ events). Details as in (B). (E) Percentage of cross-reactive CD8^+^ T cells in KK10 non-responders. Details as in (C). (F) Proviral sequence variation in KK10 responders and KK10 non-responders. Common variants are highlighted in red. Horizontal bars indicate mean values (A, C, and E). Data are representative of at least 3 independent experiments (A–E).

To confirm these findings and assess cross-reactivity among epitope forms, we visualized antigen-specific CTLs directly *ex vivo* using HLA-B^∗^27:05-peptide dextramers in conjunction with flow cytometry. KK10-specific CTL populations were observed in six patients (termed KK10 responders) and varied in magnitude from approximately 1%–11% of all CD8^+^ T cells (Figures 5A and 5B). CTL populations specific for other KK10 epitope forms were identified in the remaining four patients (KK10 non-responders) (Figures 5A and 5B). The biggest CTL populations in KK10 responders were directed against KK10, KI11, and KM14, and the biggest CTL responses in KK10 non-responders were directed against KL8 and KM14. These differences between groups could be associated with distinct patterns of proteasomal digestion, as suggested previously (Tenzer et al., 2009, 2014) and by the data shown in Figure 1.

A similar pattern of flow cytometric dextramer staining was observed for each KK10 epitope form (Figure 5B). CTL populations identified with the KK10, KI11, and KM14 dextramers were typically discrete, whereas CTL populations identified with the KL8 and KR13 dextramers were usually less discrete (Figure 5B). Fluorescence intensity also varied with specificity. For example, the brightest stains were observed with the KK10 dextramer, whereas the most heterogeneous stains were observed with the KL8 and KR13 dextramers (Figure 5B). This latter observation suggests that CTL responses directed against KL8 and KM13 incorporated lymphocytes carrying TCRs with particularly diverse avidities compared with T cells recognizing KK10, KI11, and KM14 (Betts et al., 2004).

KI11-specific CTLs were more cross reactive than other epitope-form-specific CTLs (Figures 5C–5E). In KK10 responders, the mean cross-reactivity level was 16% of all KI11 dextramer^+^ cells, and in KK10 non-responders, it was 5.6% of all KI11 dextramer^+^ cells (p = 0.032, Mann-Whitney *U* test) (Figures 5C–5E). While KI11-specific CTLs among KK10 responders predominantly cross-recognized KK10 (Figure 5C), among KK10 non-responders, KI11-specific CTLs predominantly cross-recognized KL8 (Figure 5E). Other epitope-form-specific CTLs also predominantly cross-recognized KL8 in KK10 non-responders, suggesting a common mode of TCR engagement via the conserved N-terminal K_1_R_2_W_3_ motif. In addition, KI11-specific CTLs in both patient groups frequently cross-recognized KR13 or KM14, but CTL cross-recognition between KR13 and KM14 was rare, suggesting that TCR docking sites were shared between KI11 and KR13 and between KI11 and KM14.

To evaluate the biological relevance of these observations, we isolated proviral DNA from KK10 responders and KK10 non-responders and sequenced the epitope-containing region of p24 Gag. The escape mutations R132K and L136M predominated in KK10 responders (p < 0.0001 for each mutation compared with KK10 non-responders, Fisher's exact test) (Figure 5F). In contrast, an upstream epitope-processing mutation (N120S) (Tenzer et al., 2014) predominated in KK10 non-responders (p = 0.0486 compared with KK10 responders, Fisher's exact test), invariably linked to the WT KK10 sequence (Figure 5F). The N120S substitution is a *bona fide* escape mutation because it abolishes the production of KL8, KK10, and KR13 (Tenzer et al., 2014). It may also impose structural constraints that impede the accumulation of intraepitope mutations, such as R132K and L136M.

Collectively, these results demonstrated a link between CTL specificity and the intrahost HIV-1 evolution.

### Truncated KK10 epitope forms interfere with CTL recognition of KK10

Because KK10 minitopes were poorly immunogenic, we hypothesized that these epitope forms could compete for binding to HLA-B^∗^27:05 in the ER. Such effects might limit the number of immunogenic KK10 epitope forms on the cell surface. To assess the functional consequences of epitope-form competition, we examined how CTL recognition of KK10 was affected by truncated epitope forms at relative concentrations similar to those generated by the proteasome (Figures 1B–1D).

**Figure 6:**
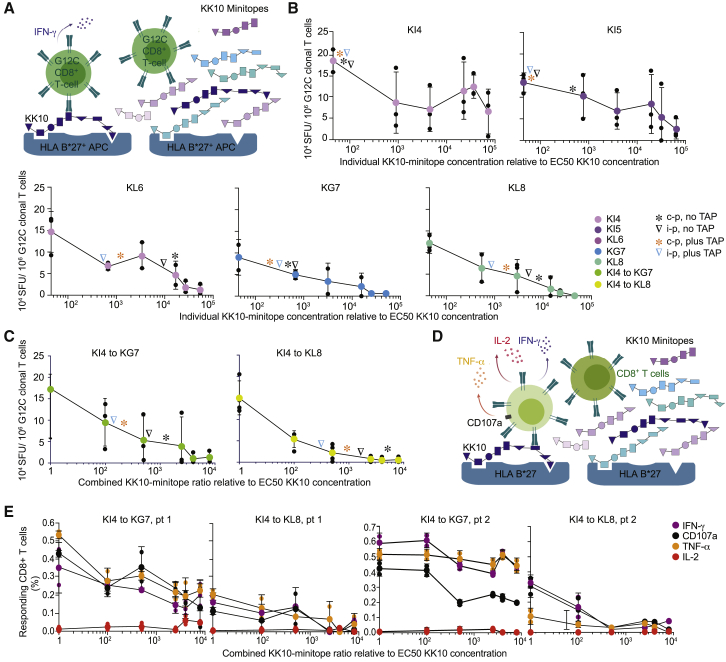
Inhibitory effects of KK10 minitopes on the functional reactivity of KK10-specific CTLs (A) Outline of the IFN-γ ELISpot competition experiment with the KK10-specific CTL clone G12C and Tap-deficient HLA-B^∗^27:05-transfected antigen-presenting cells (APCs). (B) IFN-γ release by G12C in response to KK10 at the half maximal effective concentration (EC_50_) molar concentration in the presence of individual KK10 minitopes at molar ratios of 1, 10, 50, 250, 400, and 800 relative to KK10. The corresponding molar ratios after processing (4 h) by constitutive proteasomes (c-p) or immunoproteasomes (i-p) are indicated by asterisks or inverted triangles, respectively. Non-TAP-adjusted ratios are shown in black. TAP-adjusted ratios are shown in orange (c-p) or blue (i-p). ELISpot data are shown as spot-forming units (SFUs) per 10^6^ cells. Data were concatenated from 3 independent experiments (black dots). Colored dots indicate mean values. Error bars indicate SD. (C) IFN-γ release by G12C in response to KK10 at the EC_50_ molar concentration in the presence of multiple KK10 minitopes at molar ratios of 1, 10, 50, 250, 400, and 800 relative to KK10. Details as in (B). (D) Outline of the intracellular cytokine staining (ICS) competition experiment with CD8^+^ T cells from HLA-B^∗^27:05^+^ patients. (E) Mobilization of CD107a (black) and production of IFN-γ (purple), TNF-α (orange), and interleukin (IL)-2 (red) by CD8^+^ T cells in response to KK10 at the EC_50_ molar concentration in the presence of multiple KK10 minitopes at molar ratios of 1, 10, 50, 250, 400, and 800 relative to KK10. Each function was measured by flow cytometry as a percentage of the total CD8^+^ T cell population. Data were concatenated from 3 or 4 independent experiments (black dots). Colored dots indicate mean values. Error bars indicate SD.

In competition IFN-γ ELISpot assays (Figure 6A), a previously described KK10-specific CTL clone (G12C) (Ladell et al., 2013) became progressively less responsive at progressively higher concentrations of each truncated epitope form (Figure 6B). The inhibitory effects of KI4 and KI5 were less striking than those of the longer minitopes, likely reflecting lower binding affinities for HLA-B^∗^27:05. Inhibition of the G12C response by a combination of minitopes was likewise more obvious in the presence of KL8 (Figure 6C). Comparison of these results with the processing data revealed that proteasomal digestion produced enough KL6 and KL8 to more than halve the G12C response to KK10 and enough KL6 and KL8 in combination with other epitope forms to inhibit the G12C response to KK10 by ≥70% (Figures 6B and 6C). In contrast, the G12C response was enhanced in a concentration-dependent manner by the addition of KR13 or KM14, and these responses were inhibited by the addition of KK10, suggesting preferential recognition of the longer epitope forms (Figure S7).

In competition intracellular cytokine staining (ICS) assays performed directly *ex vivo* (Figure 6D), a combination of KI4, KI5, KL6, and KG7 inhibited the KK10-specific CTL response less than a combination of KI4, KI5, KL6, KG7, and KL8 (Figure 6E). These inhibitory effects were observed across different effector functions, including the mobilization of CD107a and the production of IFN-γ and TNF-α, and were consistent with the G12C data (Figure 6C).

Collectively, these results demonstrated that truncated KK10 epitope forms, and particularly the longer minitopes, were able to compete for binding to HLA-B^∗^27:05 and inhibit CTL responses to KK10 and its extended epitope forms *in vitro*, consistent with a previously unrecognized mechanism of viral escape *in vivo*.

### KIR3DL1 binding and the effect of mutations depend on KK10 epitope form

Binding of the inhibitory KIR receptor, KIR3DL1, to HLA-B^∗^27:05-KK10 is associated with a weak but consistently beneficial effect on immune control of HIV-1, while binding to HLA-B^∗^57^∗^01 confers more robust control (Boelen et al., 2018; Martin and Carrington, 2013; Martin et al., 2007; Stewart-Jones et al., 2005a). Previous work has shown that KIR3DL1 binds HLA-B^∗^27:05-peptide complexes with a footprint that includes residues 77_B27_, 81_B27_, 84_B27_, and 88_B27_ and the C terminus of the peptide, including the amino acid residues at positions 7 (or C–2) and 8 (or C–1) (Peruzzi et al., 1996; Stewart-Jones et al., 2005a). This binding mode suggests that direct interactions are unlikely between KIR3DL1 and HLA-B^∗^27:05 in complex with KI4, KI5, KL6, or KG7. However, these truncated epitope forms might indirectly limit interactions with KIR3DL1 by outcompeting longer epitope forms for binding to HLA-B^∗^27:05.

**Figure 7:**
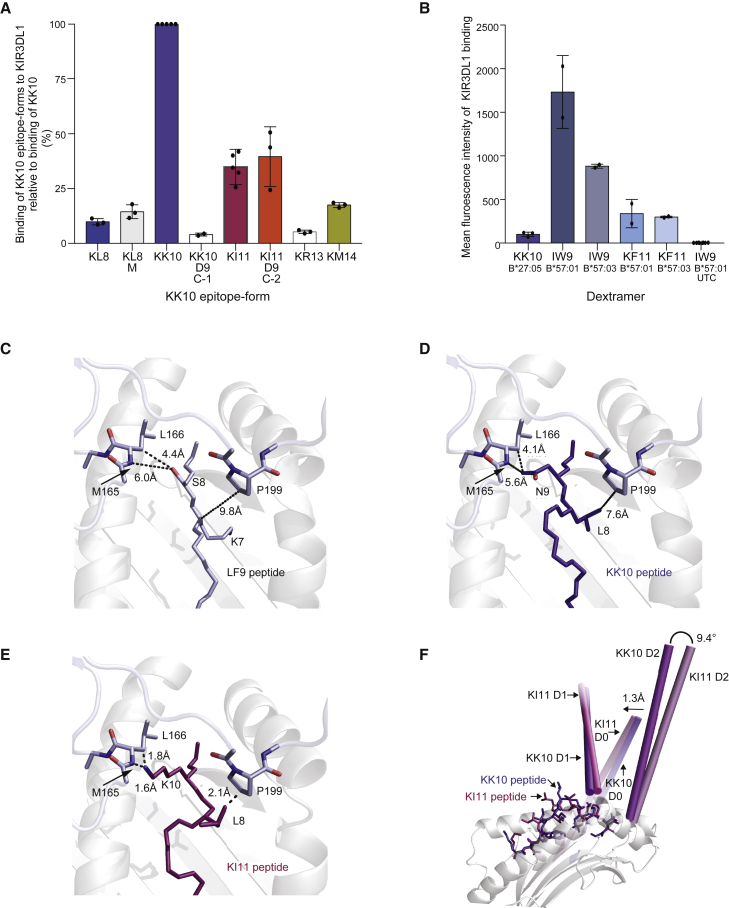
KIR3DL1 binding to KK10 epitope forms complexed with HLA-B^∗^27:05 and modeling of KIR3DL1 interactions (A) HLA-B^∗^27:05-peptide dextramer staining of a BAF cell line stably transfected with KIR3DL1^∗^001. Percentages are shown relative to KK10. The L136M mutation favored the production of KK10 minitopes and was therefore only tested in the KL8 epitope form (KL8-M). KIR3DL1 binding was reduced for all epitope forms relative to KK10 (p < 0.0001, one-way ANOVA with Dunnett's test for multiple comparisons). (B) Mean fluorescence intensity of KIR3DL1 binding to HLA-B^∗^27:05-KK10 and HLA-B57:01-IW9, HLA-B57:03-IW9, HLA-B57:01-KF11, and HLA-B57:03-KF11. KIR3DL1 binding was reduced for HLA-B^∗^27:05-KK10 relative to all other dextramers (p < 0.001 one-way ANOVA with Dunnett's test for multiple comparisons). Black dots represent independent experiments. (A and B) Error bars indicate SD. (C) Interaction between KIR3DL1 and the LF9 peptide in the 3VH8-HLA-B^∗^57:01 complex (Vivian et al., 2011) (PDB: 3VH8) showing a VDW contact between S8_peptide_ and L166_KIR3DL1_ (dotted line, 4.4 Å). This residue also had a water-mediated interaction with KIR3DL1. (D) *In silico* modeling of the KIR3DL1 interaction with HLA-B^∗^27:05-KK10 using LSQ alignment of Cα atoms from residues 1–180 of HLA-B^∗^27:05. The image shows that a VDW contact could be formed between N9_KK10_ and L166_KIR3DL1_ (dotted line, 4.1 Å). (E) *In silico* modeling of the KIR3DL1 interaction with HLA-B^∗^27:05-KI11 as in (D). The protruding K10_KI11_ might interact with E282_KIR3DL1_ and/or form a VDW contact with L166_KIR3DL1_ (dotted line, 1.8 Å) and/or a hydrogen bond contact with M165_KIR3DL1_ (dotted line, 1.6 Å). This protrusion could “push” KIR3DL1 away and/or disrupt the ordered water molecules between KIR3DL1 and HLA-B^∗^27:05. (F) *In silico* modeling of the shift in the KIR3DL1 D0, D1, and D2 domains after binding to HLA-B^∗^27:05-KK10 (blue purple) or HLA-B^∗^27:05-KI11 (red purple). The largest shift was 1.3 Å in the D1 domain, and the position of KIR3DL1 was altered by up to 9.4° in the D0 domain. The KIR3DL1 domains are represented by sticks. Models were generated using HADDOCK.

We measured KIR3DL1 binding to KK10 epitope forms complexed with HLA-B^∗^27:05 using the corresponding dextramers and a BAF cell line stably transfected with KIR3DL1^∗^001 (Stewart-Jones et al., 2005a) (Figure 7A). Similar experiments were performed using HLA-B^∗^57:01 and HLA-B^∗^57:03 dextramers complexed with the p24 Gag epitopes IW9 or KF11 to quantify protective KIR3DL1 interactions associated with elite control of HIV-1 (Carrington and Alter, 2012; Song et al., 2014) (Figure 7B). As expected, KIR3DL1 bound strongly to HLA-B^∗^57:01/03, especially in complex with IW9 (Figure 7B). In contrast, a substantially weaker interaction was observed between KIR3DL1 and HLA-B^∗^27:05-KK10 (Figure 7B).

The interaction between KIR3DL1 and HLA-B^∗^27:05-KI11 was approximately 50% weaker in terms of fluorescence intensity than the interaction between KIR3DL1 and HLA-B^∗^27:05-KK10 (Figure 7A). Further reductions were observed with other epitope forms (KM14 > KL8 > KR13) (Figure 7A). These results might be explained by the unstable conformation of KL8 and steric interference arising from the central peptide bulges in KR13 and KM14. Incorporation of the L136M mutation into KL8 marginally increased binding to KIR3DL1. *In silico* modeling suggested that KIR3DL1 docked onto HLA-B^∗^27:05-KK10 in a manner similar to the reported interaction with HLA-B^∗^57:01-3VH8 (Vivian et al., 2011) (Figures 7C and 7D), whereas the interaction with HLA-B^∗^27:05-KI11 likely would be hampered by the protruding K10_KI11_ (Figure 7E). The model suggested that the protruding K10_KI11_ would alter the position of the D1 domain, leading to shifts in all three KIR domains that would change and/or disrupt contacts across the interface (Figure 7F).

The N139D mutation predominated in KK10 non-responders (Figure 5F). To examine the effect of this substitution on KIR3DL1 recognition, we measured KIR3DL1 binding using HLA-B^∗^27:05 dextramers complexed with KK10-D9 (C–1) or KI11-D9 (C–2). The D9 substitution in KK10 dramatically reduced binding to KIR3DL1 (Figure 7A), and modeling suggested that the negatively charged D9_peptide_ disrupted hydrogen bonds between N9_KK10_ and E76_B27_ and N77_B27_ in the α1_B27_ helix. The presence of a negative charge at the C–1 position has been shown previously to abolish KIR3DL1 recognition (Brackenridge et al., 2011; O'Connor et al., 2015). In contrast, KIR3DL1 bound equivalently to HLA-B^∗^27:05-KI11 and HLA-B^∗^27:05-KI11-D9 (Figure 7A). This experimental observation and modeling suggested that the D9_peptide_ side chain adopted a similar conformation to the N9_KI11_ side chain, which pointed toward the binding groove of HLA-B^∗^27:05 (Figure 4A). Alternatively, the D9 substitution induced subtle changes in peptide conformation, which differed in the context of KK10 and KI11 (Iversen et al., 2006).

Collectively, these findings showed that KIR3DL1 bound less avidly to HLA-B^∗^27:05 complexed with any KK10 epitope form than to HLA-B^∗^57:01/03 complexed with IW9 or KF11. Moreover, KIR3DL1 bound less well to all immunogenic non-10-mer KK10 epitope forms than to the optimal KK10 epitope form. Notably, if the intraepitope N139D mutation was present in the KK10 epitope, the binding to KIR3DL1 was markedly reduced, whereas no significant reduction was observed when the mutation was present in the KI11 epitope form. Together, the processing of multiple epitope forms and intraepitope mutations resulted in a relative decrease in the binding strength between KIR3DL1 and the HLA-B^∗^27:05-peptide complex, which likely decreases inhibitory KIR signaling and the NK response to HIV-1 infection (Boudreau et al., 2016; Saunders et al., 2016). These results are consistent with the absent/weak and strong biological effects of inhibitory KIR interactions with HLA-B^∗^27:05 and HLA-B^∗^57:01/03, respectively, in HIV-1 containment (Martin and Carrington, 2013; Martin et al., 2007).

## Discussion

Here, we found that the immunodominant KK10 region of p24 Gag was naturally processed into multiple epitope forms, many of which bound TAP and HLA-B^∗^27:05. Structural analyses revealed distinct and shared molecular features that affected immune recognition of these epitope forms via KIR3DL1 and TCRs. In addition, common intraepitope CTL escape mutations, including R132K (Feeney et al., 2004; Goulder et al., 1997) and L136M (Almeida et al., 2007; Ammaranond et al., 2005; Feeney et al., 2004; Goulder et al., 1997; Ladell et al., 2013; Schneidewind et al., 2007, 2008), skewed proteasomal activity to favor the production of KK10 minitopes, which competitively limited interactions with KIR3DL1 and TCRs. Even very short minitopes, such as KI4 and KI5, incorporated optimal amino acids that allowed for binding to TAP (Momburg et al., 1994a, 1994b; van Endert, 2011; van Endert et al., 1995). However, although TAP binding is the rate-limiting step, translocation into the ER of such short peptides has not been proved nor disproved experimentally (Gubler et al., 1998). Processing of multiple epitope forms, including the generation of inhibitory minitopes, was therefore identified as a mechanism of viral escape from CTL responses and inhibitory KIR binding in the context of the protective allotype HLA-B^∗^27:05.

In patients with KK10-specific CTL responses, the intraepitope substitutions R132K and L136M predominated at the level of proviral DNA. These mutations are known to impose fitness costs on HIV-1 (Schneidewind et al., 2008). A KK10-specific CTL selection pressure was therefore apparent in these patients, despite the relatively low amount of KK10 produced following processing of the HIV-1 subtype B WT sequence. This observation could be explained by the fact that very few cell surface recognition events are required to activate CTLs (Purbhoo et al., 2004). That the intraepitope CTL escape mutation, L136M, skewed processing toward exclusive production of KK10 minitopes would further aid viral escape and might help explain why cross-reactive CTLs ultimately fail to prevent disease progression in HLA-B^∗^27:05^+^ individuals with enhanced immune control of HIV-1 (Ladell et al., 2013). The R132K mutation abrogated binding to HLA-B^∗^27:05, which resulted in viral escape regardless of epitope form. In patients without a KK10-specific CTL response, the extraepitope substitution N120S predominated at the level of proviral DNA. This mutation was invariably associated with an intact KK10 sequence and likely disrupted the production of multiple epitope forms to prevent immune recognition by cross-reactive or variant-specific TCRs (Tenzer et al., 2014).

KIR3DL1 bound less well to HLA-B^∗^27:05 complexed with KK10 than to HLA-B^∗^57:01/03 complexed with the protective epitopes IW9 or KF11, and binding decreased further when considering shorter or longer KK10 peptide variants. Strong inhibitory KIR3DL1 ligation has been demonstrated to improve NK cell activation and antibody-dependent cellular cytotoxicity (ADCC), inhibit HIV-1 replication in autologous infected CD4+ T cells, and enhance survival of CD8+ T cells in viral infection (Boelen et al., 2018; Boudreau et al., 2016; Song et al., 2014). The HIV-1 Nef protein selectively downregulates HLA-A and -B, which aid viral escape from the CTL response but render the cells vulnerable to NK lysis. Differential epitope peptide processing, and the competition between immunogenic and non-immunogenic epitope forms within the ER, would likely decrease inhibitory KIR3DL1 signaling in HIV-infected patients with HLA-B^∗^27:05 and might therefore provide a viral escape mechanism.

The effect of the intraepitope mutation (N139D) on KIR3DL1 binding differed considerably depending on the length of the epitope form containing the mutation as binding was reduced in the context of KK10 and unaffected in KI11. As far as we know, this phenomenon has not been previously reported and underscores that epitope-processing patterns need to be considered when assessing the effects of mutations on binding to KIR3DL1.

The epitope form KI11 was frequently cross recognized in patients with or without a contemporaneous response to KK10. This observation could be explained by the conserved N- and C-terminal motifs and the distinct central peptide bulge in HLA-B^∗^27:05-KI11. The latter appeared more stable than the corresponding peptide bulges in HLA-B^∗^27:05-KR13 and HLA-B^∗^27:05-KM14, potentially enabling more productive interactions with cross-reactive TCRs. Comparable peptide bulges have been observed previously in complexes with other HLA class I allotypes (Hillig et al., 2004) (Stewart-Jones et al., 2005b) (Tynan et al., 2005) (Probst-Kepper et al., 2004) (Bade-Doding et al., 2011).

The poor definition and variable fluorescence of KL8-specific CTL populations suggested cross-recognition via an array of clonotypes, potentially reflecting the availability of distinct structural features in the antigenic complex, such as the conserved N-terminal K_1_R_2_W_3_ motif and the exposed C-terminal residues in the partially bound conformation, which might be forced into the less common extended conformation after engagement with particular TCRs. This interpretation contrasted with the single recognition structure model (Bordner, 2010) and was somewhat analogous to earlier observations in the murine I-E^k^ system (Schmitt et al., 1998).

Peptide competition has been mooted as a therapeutic approach to inhibit or silence errant immune responses in autoimmunity (Lock et al., 1991). Our results provide an indirect proof of concept that such a strategy could work *in vivo* because the production of inhibitory peptides was favored as a means of viral escape by natural selection. HLA-B^∗^27:05 confers a strong inherited risk to several autoimmune diseases, e.g., ankylosing spondylitis, juvenile arthritis, reactive arthritis, and acute anterior uveitis. Although treatment of these diseases has been revolutionized with the advent of tumor necrosis factor (TNF) inhibitors, many patients do not respond or show loss of clinical response over time. Therefore, new therapeutic modalities are needed. Our study indicates that it might be possible to design inhibitory peptides, or perhaps short versions of key disease-associated peptides, that bind with high affinity to the HLA-B^∗^27:05 peptide-binding cleft and hamper pathogenic T cell responses. Innovative vehicles might be required to deliver such peptides to disease sites, e.g., exosomes (Barile and Vassalli, 2017), although recent immunotherapy with peptides designed to bind the HLA binding cleft to modify type 1 diabetes causing T responses showed promise following intravenous administration (Alhadj Ali et al., 2017).

It will be important to confirm and extend the findings reported here across other antigenic regions of HIV-1 and possibly other viruses. As the epitope-specific CTL response is influenced by the abundance (Schmidt et al., 2012; Tenzer et al. 2009, 2014) and length of the specific epitope peptide (Iversen et al., 2006), it will likely be possible to use the knowledge gained from antigen-processing analyses of HIV-1 to “reverse engineer” and create artificial HIV-1 vaccine inserts that increase the processing of specific, highly immunogenic forms of selected epitopes.

### Limitations of the study

We determined the TAP affinities of the KK10 epitope peptides, as TAP binding is the rate-limiting step for peptide transport into the ER (Gubler et al., 1998). However, it cannot be ruled out that translocation efficiency might differ. The current peptide elution technology prevented simultaneous analyses of the peptide repertoire in the ER and the cell surface. As a result, the intracellular competition between the peptides had to be analyzed through peptide competition experiments at the cell surface.

## STAR★Methods

### Key resources table

**Table:** 

REAGENT or RESOURCE	SOURCE	IDENTIFIER
**Antibodies**		

anti-human-CD4–APC-eFluor780	eBioscience	Cat#47-0049-42 RRID:AB_1272044
anti-human-CD8–PerCP	BD Biosciences	Cat#347314 RRID:AB_400280
anti-human-CD28	BioLegend	Cat# 302901 RRID:AB_314303
anti-human-CD49d	BioLegend	Cat# 304301 RRID:AB_314427
anti-human-CD107a-FITC	BioLegend	Cat#328605 RRID:AB_1186058
anti-human-IFN-γ–PE-Cy7	BioLegend	Cat#502527 RRID:AB_1626154
anti-human-TNF-α–APC	BioLegend	Cat# 502913 RRID:AB_315265
Anti-human-IL-2-PE	BioLegend	Cat# 500306 RRID:AB_315093
anti-human CD3-ECD	Beckman Coulter	Cat# IM2705U RRID:AB_130860
Live Dead Violet Viability Dye	ThermoFisher	Cat#L34955
Bacterial and virus strains		
*E*. *coli* DH5a	ThermoFisher	Cat#18258012
One Shot™ BL21(DE3)pLysS *E. coli*	ThermoFisher	Cat#C606003

**Biological samples**		

HLA B^∗^27+ patient samples	Department of Infectious Diseases, Rigshospitalet, Denmark	Patient 1 to 10

**Chemicals and recombinant proteins**		

Trypsin EDTA	SigmaAldrich	Cat#59428C
Recombinant HLA B^∗^27:05	This paper	N/A
Human IL-2	SigmaAldrich	Cat# I17002
Human IL-15	SigmaAldrich	Cat# I8648
Brefeldin A	BioLegend	Cat#420601
Monensin	BioLegend	Cat#420701
Phorbol myristate acetate	SigmaAldrich	Cat#P8139
Ionomycin	SigmaAldrich	Cat#I9657
Mouse IL-3	BioLegend	Cat#575502
HLA-B^∗^27^∗^05-PE Dextramers	Immudex	Custom
HLA-B^∗^27^∗^05-APC Dextramers	Immudex	Custom
KRWI (KI4)	Schafer-N	Custom
KRWII (KI5)	Schafer-N	Custom
KRWIIL (KL6)	Schafer-N	Custom
KRWIILG (KG7)	Schafer-N	Custom
KRWIILGL (KL8)	Schafer-N	Custom
KRWIILGLNKI (KI11)	Schafer-N	Custom
KRWIILGLNKIVR (KR13)	Schafer-N	Custom
KRWIILGLNKIVRM (KM14)	Schafer-N	Custom

**Deposited data**		

Crystal Structure Of HLA-B^∗^2705 Complexed With the self-Peptide TIS from EGF-response factor 1	Hulsmeyer et al., 2005	PDB ID: 1W0V
Crystal structures and KIR3DL1 recognition of three immunodominant viral peptides complexed to HLA-B2705.	Stewart-Jones et al., 2005a	PDB ID: 2BSS
KIR3DL1 in complex with HLA-B^∗^5701	Vivian et al., 2011	PDB ID: 3VH8
HLA-B^∗^27:05 presenting an HIV-1 6mer peptide	This Paper	PDB ID: 6VQZ
HLA-B^∗^27:05 presenting an HIV-1 7mer peptide	This Paper	PDB ID: 6VQY
HLA-B^∗^27:05 presenting an HIV-1 8mer peptide	This Paper	PDB ID: 6VQD
HLA-B^∗^27:05 presenting an HIV-1 11mer peptide	This Paper	PDB ID: 6VPZ
HLA-B^∗^27:05 presenting an HIV-1 13mer peptide	This Paper	PDB ID: 6VQE
HLA-B^∗^27:05 presenting an HIV-1 14mer peptide	This Paper	PDB ID: 6VQ2

**Experimental models: Cell lines**		

T2	ATCC	Cat#CRL-1992
Ba/F3	DSMZ	Cat# ACC 300

**Oligonucleotides**		

GAG1 (round 1 (R1)) 728–751 (HIV numbering)	Chang et al., Sci. Rep, https://doi.org/10.1038/srep11253	Custom Synthesis
GAG3 (R1) 1941–1916 (HIV_HXB2_ numbering)	Chang et al., Sci. Rep, https://doi.org/10.1038/srep11253	Custom Synthesis
GAG2 (R2) 763-788 (HIV_HXB2_ numbering)	Chang et al., Sci. Rep, https://doi.org/10.1038/srep11253	Custom Synthesis
GAG4 (R2) 1911–1884 (HIV_HXB2_ numbering)	Chang et al., Sci. Rep, https://doi.org/10.1038/srep11253	Custom Synthesis

**Recombinant DNA**		

HLA-B25:05-pGMT7 plasmid	Simon Kollnberger (co-author), https://doi.org/10.1002/eji.200425724 2005	Custom Synthesis

**Software and algorithms**		

ChimeraX	Pettersen et al., 2021	https://www.cgl.ucsf.edu/chimerax/
Chimera v1.1.3	Pettersen et al., 2004	https://www.cgl.ucsf.edu/chimera/
Phenix v1.16	Liebschner et al., 2019	www.phenix-online.org
Coot v0.9.5	Emsley et al., 2010	https://www2.mrc-lmb.cam.ac.uk/personal/pemsley/coot/
PyMOL v2.3	The PyMOL Molecular Graphics System, Version 2.3 Schrödinger, LLC	https://pymol.org/2/
PISA	European Bioinformatics Institute	https://www.ebi.ac.uk/pdbe/prot_int/pistart.html
CCP4 v7.1	Winn et al., 2011	https://www.ccp4.ac.uk
XDS	Kabsch, 2010	https://xds.mr.mpg.de
HADDOCK v2.4	de Vries et al., 2007; Dominguez et al., 2003	https://wenmr.science.uu.nl/haddock2.4/
Summit v4.3	Beckman Coulter (Dako Cytomation)	https://www.beckman.com
Prism v9.0	Graphpad	https://www.graphpad.com/scientific-software/prism/
R v4.0.5	R Core Team., 2020	https://www.r-project.org
Stat Trek		https://stattrek.com

### Resource availability

#### Lead contact

Further information and requests for resources and reagents should be directed to and fulfilled by the lead contact, Astrid K.N. Iversen (astrid.iversen@ndcn.ox.ac.uk).

#### Materials availability

This study did not generate new unique reagents. The structures were deposited in the Protein Data Bank (PDB: 6VQZ for HLA-B^∗^27:05-KL6; PDB: 6VQY for HLA-B^∗^27:05-KG7; PDB: 6VQD for HLA-B^∗^27:05-KL8; PDB: 6VPZ for HLA-B^∗^27:05-KI11; PDB: 6VQE for HLA-B^∗^27:05-KR13; and PDB: 6VQ2 for HLA-B^∗^27:05-KM14).

### Experimental models and subject details

#### Patients and HLA genotyping

Patients with chronic HIV-1 infection were recruited from the Department of Infectious Diseases, Rigshospitalet, Denmark. All patients were female and were between 24 and 43 years of age. All patients gave written informed consent and were studied according to the regulations of the Danish Board of Medical Ethics. HLA genotyping was performed using a multiplex PCR (Dynal Biotech). Ten patients were found to carry the HLA-B^∗^27:05 allele (Table S3).

### Method details

#### Peptide synthesis and purification

Peptides were synthesized using an MK-IV peptide synthesizer (Schafer-N) and separated at > 98% purity using a JupiterProteo C12 column (Phemomenex)(Tenzer et al., 2009).

#### Proteasome purification and *in vitro* digestions

Constitutive proteasomes and immunoproteasomes (20S) were purified from LCL721.174 and LCL721 EBV-transformed human B cell lines, respectively (Tenzer et al., 2004). *In vitro* digestions with purified constitutive proteasomes and immunoproteasomes were performed at a substrate:enzyme ratio of 1,000:1 for 1, 2, 4, or 6 h. All peptide digestions in one experimental set were performed on the same day. Data were reported after 4 h, at which time < 40% of the initial substrate was degraded in all experiments, allowing the detection of all cleavage products while minimizing the effects of repeated proteasomal digestions.

#### Analysis of peptide digests by mass spectrometry

Peptide digests were analyzed via capillary liquid chromatography (LC) using a Waters NanoAcquity UPLC System (Waters) and via mass spectrometry (MS) using a Q-Tof Premier Mass Spectrometer (Waters) (Tenzer et al., 2009). Each sample was analyzed in triplicate. LC-MS^E^ data were processed using the ProteinLynx Global Server (Waters). The mass error tolerance values were typically < 5 ppm. Mass spectrometric fragment intensity was used as a surrogate marker for quantity(Tenzer et al., 2009).

#### TAP-peptide binding assays and ERAP1/2 digestions

TAP-peptide competitive binding assays and normalization of results, and ERAP1/2 digestions were performed as described previously (Tenzer et al., 2009).

#### Expression, purification, and crystallization of HLA-B^∗^27:05-peptide complexes

HLA-B^∗^27:05 heavy chain and β2M proteins were expressed and purified as described previously (Burgess, 2009; Tenzer et al., 2009) and refolded with peptide using a rapid dilution method (Tsumoto et al., 2003). Heterotrimeric complexes were purified via size exclusion using a HiLoad 26/600 Superdex 75 pg column fitted to an AKTA Purifier FPLC System (GE Healthcare). Eluent fractions corresponding to absorbance peaks were examined via SDS-PAGE. Heterotrimeric complexes were then purified again via size exclusion using a Superdex 75 10/300 GL column fitted to an AKTA Purifier FPLC System (GE Healthcare). Correctly refolded heterotrimers were concentrated to > 10 mg/mL for crystallization trials using Amicon Ultra-0.5 Centrifugal Filter Units with Ultracel-10 Membranes (Merck Millipore). Optimal protein concentrations were determined for each complex using a PCT Pre-Crystallization Test (Hampton Research). Correctly refolded heterotrimers that did not crystallize were examined using mass spectrometry. Crystallization was carried out using the sitting-drop vapor-diffusion method. Briefly, crystallization screen reagents (Hampton Research) were dispensed into a CrystalQuick 96 Well Sitting Drop Plate (Greiner) using a Hydra eDrop II (Thermo Fisher Scientific), and 10 nL of protein was dispensed as a sitting drop in each well after mixing with 10 nL of the corresponding well buffer using a Microsys Liquid Handler (Cartesian Technologies). Crystal screens were stored at 4°C using a Protein Crystallization Imager (Formulatrix). Crystals obtained from the screens were immersed in reservoir buffer containing 20% glycerol as a cryoprotectant and vitrified at 100 K in liquid nitrogen. X-ray diffraction was carried out on beamlines at the European Synchrotron Radiation Facility, Grenoble, France (www.esrf.eu) or at the Diamond Light Source, Didcot, UK (www.diamond.ac.uk).

#### Modeling of HLA-B^∗^27:05-peptide complexes and the interaction between HLA-B^∗^27:05 and KIR3DL1^∗^001

HLA-B^∗^27:05-peptide models were constructed using molecular replacement using PDB ID: 1W0V with the original peptide was omitted (Hulsmeyer et al., 2005). Manual building and refinement were carried out using Coot (Emsley et al., 2010), Buster (Bricogne et al., 2011), and Phenix (Adams et al., 2010). Simulated annealing was performed during the initial refinement process to avoid bias from the reference model. Non-crystallographic symmetry restraints were used for the KL6 and KG7 structures, in which the asymmetric unit comprised two HLA-B^∗^27:05-peptide complexes, and these restraints were removed for the final round of refinement (Pettersen et al., 2004) (Kabsch, 2010) (Winn et al., 2011) (Pettersen et al., 2021). Model validation was carried out using MolProbity (Chen et al., 2010). Figures were prepared using the PyMol Molecular Graphics System (Schrödinger Inc.). The Adaptive Poisson-Boltzmann Solver (APBS) plugin was used to calculate electrostatic potentials for the models shown by solving the Poisson-Boltzmann equation (Baker et al., 2001). B-factors (average mean atomic displacement from the position in the refined structure) were normalized *using the HLA heavy-chain data across all models* to standardize the average B-factor of the HLA among the six structures, and peptide B-factors were compared to give a measure of relative local disorder.

The HLA-B^∗^27:05 interaction with KIR3DL1^∗^001 for the KK10 and KI11 epitopes was modeled using PDB entries 2BSS and 3VH8 for HLA-B^∗^27:05-KK10 and KIR3DL1^∗^001, respectively, along with our structure for HLA-B^∗^27:05-KI11 (PDB ID: 6VPZ). Docking was modeled using HADDOCK (de Vries et al., 2007; Dominguez et al., 2003). Restraints included all contacts at the KIR3DL1^∗^001-HLA-B^∗^57:01 interface, excluding the peptide contact at position 8 listed previously (Vivian et al., 2011) (Liebschner et al., 2019). Passive residues were defined automatically. Manual error checking was undertaken using Coot (Emsley et al., 2010), guided by the electron density submitted for the KIR3DL1^∗^001-HLA-B^∗^57:01 complex (PDB ID: 3VH8).

#### *Ex vivo* staining of PBMCs with HLA-B^∗^27:05-peptide dextramers

PBMCs were stained with LIVE/DEAD Fixable Violet (Thermo Fisher Scientific). Cells were then washed twice and incubated with HLA-B^∗^27:05-peptide dextramers as per the manufacturer's protocol (Immudex). Surface markers were identified using anti-CD3–ECD (Beckman Coulter), anti-CD4–APC-eFluor780 (eBioscience), and anti-CD8–PerCP (BD Biosciences). Cells were then washed again and fixed with 1% formaldehyde for a minimum of 1 h. Non-identical HLA-A^∗^A2:01-peptide dextramers containing an irrelevant epitope were used as gating controls. Unstained and single-stained cells were used to determine compensation values. Samples were acquired using a CyAn ADP Analyzer (Beckman Dako Cytomation), and data were analyzed using FlowJo software (Tree Star).

#### Amplification and sequencing of HIV-1 p24 Gag

DNA was extracted from approximately 10^5^ PBMCs using a Gentra Puregene Blood Core Kit A (Qiagen). PCR amplification of ∼1,200 bases spanning the HIV-1 p17–p24 region was performed as described previously (Chang et al., 2015). Amplicons were cloned using a TOPO TA Cloning Kit (Thermo Fisher Scientific) and sequenced using a 3730 DNA Analyzer (Applied Biosystems). Ten clones were analyzed per sample. Forward and reverse sequence reads were assembled and proofread using Sequencher (Gene Codes Corporation) and aligned and manually edited using MacClade (Sinauer Associates).

#### Standard and competition IFN-γ ELISpot assays

G12C cells were expanded as described previously(Iversen et al., 2006) and rested for 2 days in fresh medium supplemented with IL-2 (20 IU/mL) and IL-15 (25 ng/mL) before testing in IFN-γ ELISpot assays (Merck Millipore) (Tenzer et al., 2009, 2014). TAP-deficient T2 cells were transfected with HLA-B^∗^27:05 to generate T2-HLA-B^∗^27:05 cells (Kuipers et al., 1996). These cells were used in competition assays to minimize the potentially confounding effects of internally loaded peptides and other HLA class I and class II molecules. T2-HLA-B^∗^27:05 cells were incubated for 30 min at 37°C with various concentrations of a given epitope-form peptide, corresponding to molar ratios of 1, 10, 50, 250, 400, and 800 relative to the KK10 peptide, which was then added at the EC50 concentration (Altfeld et al., 2000). The EC50 concentration was determined in preliminary ELISpot assays as the concentration required to elicit half-maximal IFN-γ production (Tenzer et al., 2009, 2014). Samples were tested in triplicate or quadruplicate. G12C responses were expressed as spot-forming units per 10^6^ cells. Standard *ex vivo* IFN-γ ELISpot assays were performed as described previously (Tenzer et al., 2009, 2014).

#### Standard and competition ICS assays

PBMCs were stimulated in a 96-well plate with various concentrations of peptide (KK10), anti-CD28 (1 μg/mL), and anti-CD49d (1 μg/mL) for 6 h in the presence of anti-CD107a–FITC (BioLegend). Cytokine secretion was blocked with brefeldin A (5 μg/mL; BioLegend) and monensin (2 μM; BioLegend). Negative control wells contained anti-CD28 (1 μg/mL) and anti-CD49d (1 μg/mL). Positive control wells contained anti-CD28 (1 μg/mL), anti-CD49d (1 μg/mL), phorbol myristate acetate (2.5 ng/mL), and ionomycin (25 ng/mL). Cells were then stained with LIVE/DEAD Fixable Violet (Thermo Fisher Scientific), fixed/permeabilized using a Cytofix/Cytoperm Kit (BD Biosciences), and stained with anti-CD3–ECD (Beckman Coulter), anti-CD8–PerCP (BD Biosciences), anti-IFN-γ–PE-Cy7 (BioLegend), anti-TNF-α–APC (BioLegend), and anti-IL-2–PE (BioLegend). Unstained and single-stained cells were used to determine compensation values. Samples were acquired using a Cyan ADP Analyzer (Beckman Dako Cytomation), and data were analyzed using Summit software (Beckman Dako Cytomation). Competition assays were performed similarly using the truncated epitope-form peptides at molar ratios of 1, 10, 50, 250, 400, and 800 relative to the KK10 peptide, which was added at the calculated EC50 concentration after 30 min at 37°C. Control wells contained truncated epitope-form peptides alone or the KK10 peptide alone. Samples were tested in duplicate.

#### KIR3DL1^∗^001 binding assay

Parental or KIR3DL1^∗^001-transfected BA/F3 cells were expanded in the presence of mouse IL-3 (0.5 ng/mL) as described previously (Stewart-Jones et al., 2005a). Each line was stained with LIVE/DEAD Fixable Violet (Thermo Fisher Scientific). Cells were then washed twice and incubated with HLA-B^∗^27:05-peptide dextramers as per the manufacturer's protocol (Immundex). The parental line was used as a control for each condition. Samples were tested in duplicate. Cells were then washed again and fixed with 1% formaldehyde for a minimum of 1 h. Samples were acquired using a Cyan ADP Analyzer (Beckman Dako Cytomation), and data were analyzed using FlowJo software (Tree Star).

### Quantification and statistical analysis

Statistical analyses were performed using the indicated tests in Excel (Microsoft), Prism (GraphPad), R (https://www.r-project.org), or Stat Trek (https://stattrek.com). Comparisons were considered significant at P < 0.05.

## Data Availability

All data reported in this paper will be shared by the lead contact upon request. This paper does not report original code. Any additional information required to reanalyse the data reported in this paper is available from the lead contact upon request.

## Supplemental information

Document S1. Figures S1–S7 and Tables S1–S3

Document S2. Article plus supplemental information
