# Supplementary material for: Epitope length variants balance protective immune responses and viral escape in HIV-1 infection
Source: Cell Rep. 2022 Mar 1;38(9):110449. doi: 10.1016/j.celrep.2022.110449 (PMC9631117; doi:10.1016/j.celrep.2022.110449)
Supplement: Document S1. Figures S1–S7 and Tables S1–S3 [file mmc1.pdf]

Cell Reports, Volume *38*

Supplemental information

Epitope length variants

balance protective immune responses

and viral escape in HIV-1 infection

Phillip Pymm, Stefan Tenzer, Edmund Wee, Mirjana Weimershaus, Anne Burgevin, Simon Kollnberger, Jan Gerstoft, Tracy M. Josephs, Kristin Ladell, James E. McLaren, Victor Appay, David A. Price, Lars Fugger, John I. Bell, Hansjörg Schild, Peter van Endert, Maria Harkiolaki, and Astrid K.N. Iversen

Figure S1

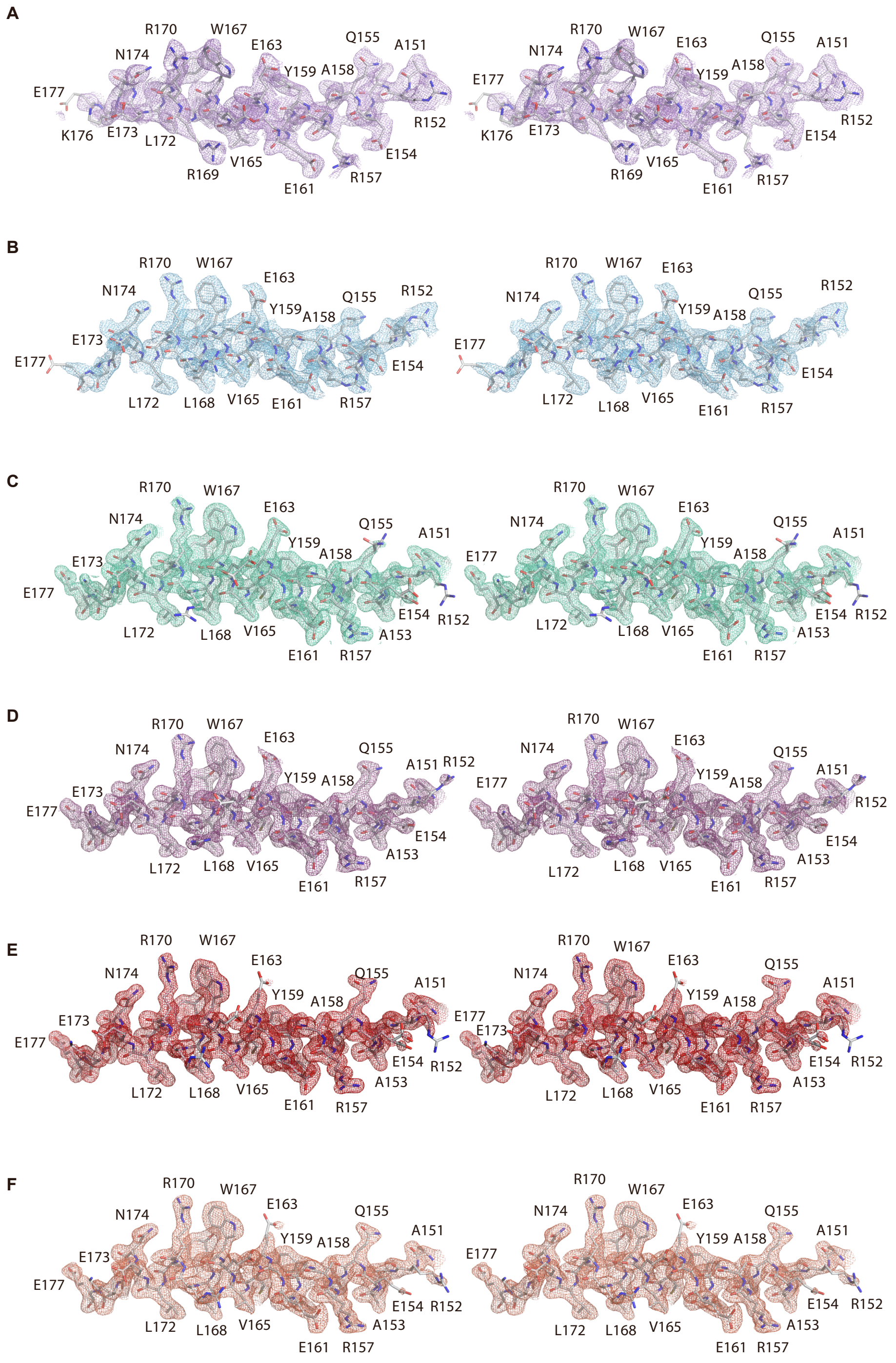

Figure S1. Stereo images of the electron density of residues 150 to 177 of the α2 helix of the HLA-B*27:05 heavy chain consisting of the following amino acid residue sequence in single-letter amino acid code: ARVAEQLRAYLEGECVEWLRR

YLENGKE, related to Figure 2. Density shown is taken from the refined 2mFo-DFc map within 1.6Å of atoms with a 1.2σ cutoff. Selected amino acid residues are labelled for HLA-B*27:05 KRWIIL (**A**), HLA-B*27:05 KRWIILG (**B**), HLA-B*27:05 KRWIILGL (**C**), HLA-B*27:05 KRWIILGLNKI (**D**), HLA-B*27:05 KRWIILGLNKIVR (**E**), HLA-B*27:05 KRWIILGLNKIVRM (**F**).

Figure S2

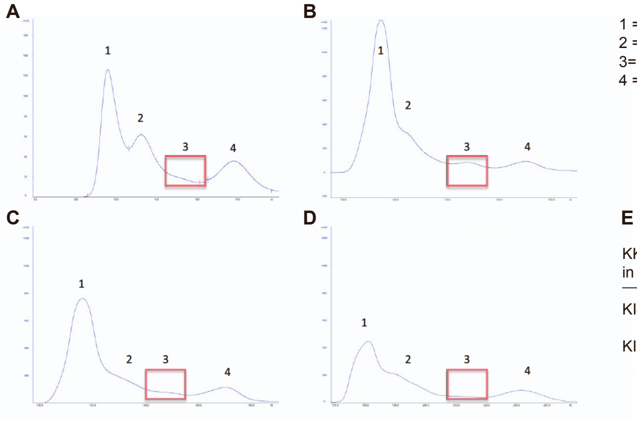

1 = Heavy aggregates
2 = HLA-B*27:05 homodimers
3= HLA-B*27:05 heterotrimer
4 = HLA-B*27:05 monomer/ β2 m homodimer

E

KK10 epitope-form in refold	Concentration
KI4	0.2 pmol/µl
KI5	10.0 fmol/µl

Figure S2. FPLC analyses of KI4, KI5, and WK8 refolds with HLA-B*27:05, related to Figures 2. (**A–E**) Fast protein liquid chromatography (FPLC) analyses of refolds containing the HLA-B*27:05 heavy chain and β2m with no peptide (control) (**A**), KI4 (**B**), KI5 (**C**), or WK8 (**D**). (**E**) Peptide concentration in the KI4 and KI5 refolds. The refolds were diluted 1:100 in 1% formic acid to free the peptides before mass spectrometry. The red box indicates the HLA-B*27:05 heterotrimer (HLA-B27:05-β2m-peptide).

Figure S3

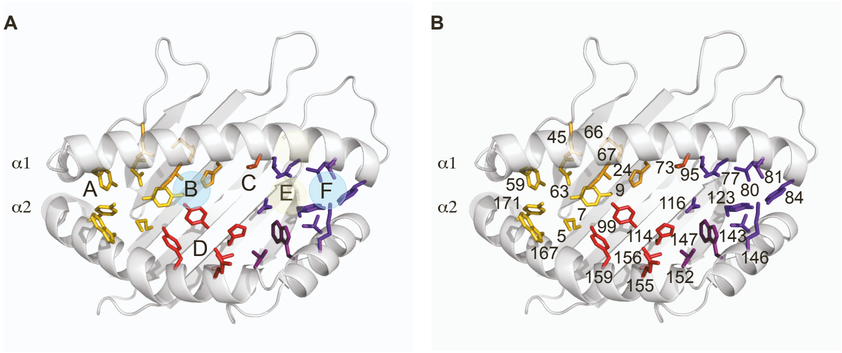

A pocket (yellow): 5, 7, 59, 63, 167, 171
B pocket (light orange): 7, 9, 24, 45, 63, 66, 67
C pocket (deep orange): 73
D pocket (red): 99, 114, 155, 156, 159
E pocket (purple): 147, 152
F pocket (blue-purple): 77, 80, 81, 84, 95, 116, 123, 143, 146

Figure S3. Overview of the HLA-B*27:05 binding groove, related to Figure 2. (**A**) Top-view of the peptide-binding groove of HLA-B*27:05 showing the A-, B-, C-, D-, E-, and F-pockets. The B- and F-pockets are indicated by light-blue disks, and the E-pocket is indicated by a light-yellow disk. (**B**) Top-view of the peptide-binding groove of HLA-B*27:05 showing the interacting amino acid residues for each pocket, colored and numbered as indicated in the key.

Figure S4

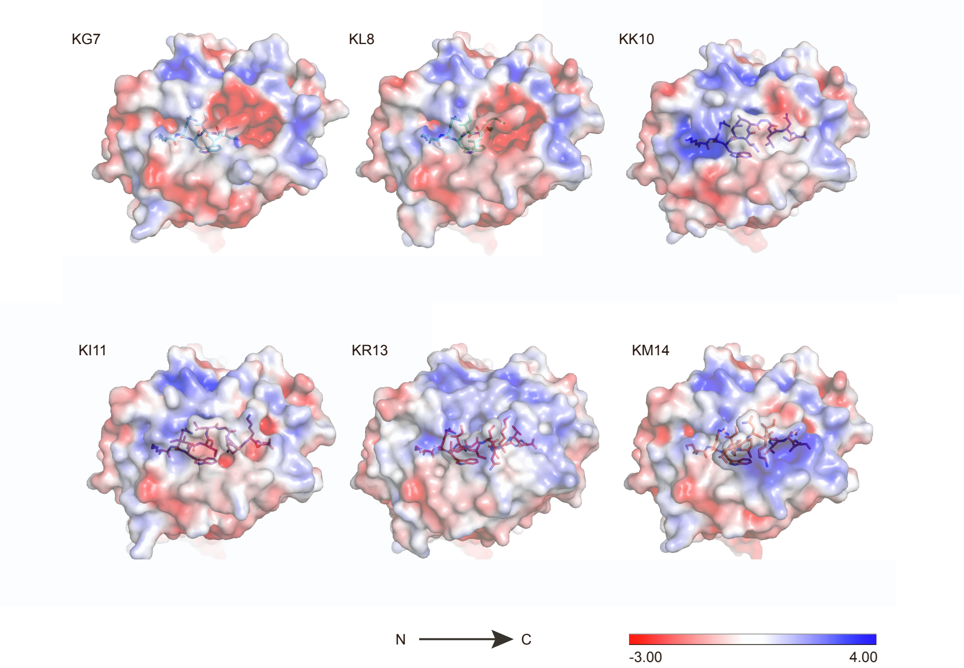

Figure S4. **Electrostatic analyses of HLA-B*27:05-peptide complexes, related to Figure 3**. Colours represent the electrostatic potential on the surface of each HLA-B*27:05-peptide complex as indicated in the key.

Figure S5

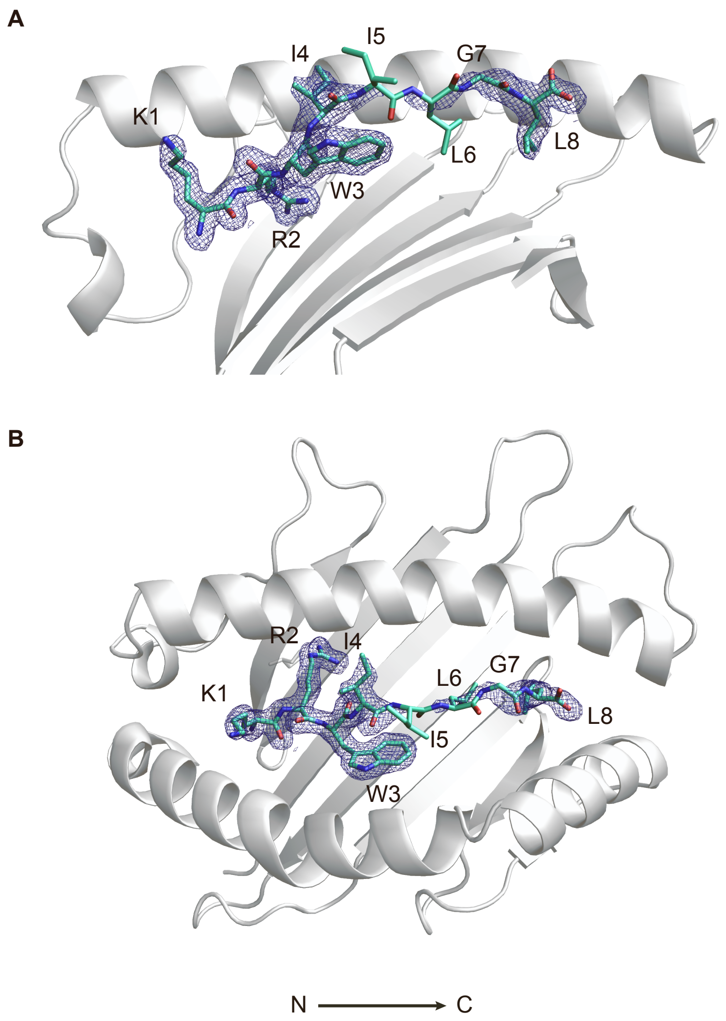

Figure S5. Images of the refined density of the KRWIILGL-SP (KL8-SP) structure, related to Figures 2 and 3. The scaled map was put through one round of refinement with the alternate model around the peptide at 1.0 sigma; (**A**) side-view and (**B**) top-view. The figures show occupancy at the C-terminus including Gly7 and a very small amount of density for positions 5 and 6. We estimate that there is ~10% occupancy for these

residues and that the better density at the C-terminus likely is explained by this position being filled with an arginine from the refolding buffer in the "normal" KL8 model.

Figure S6

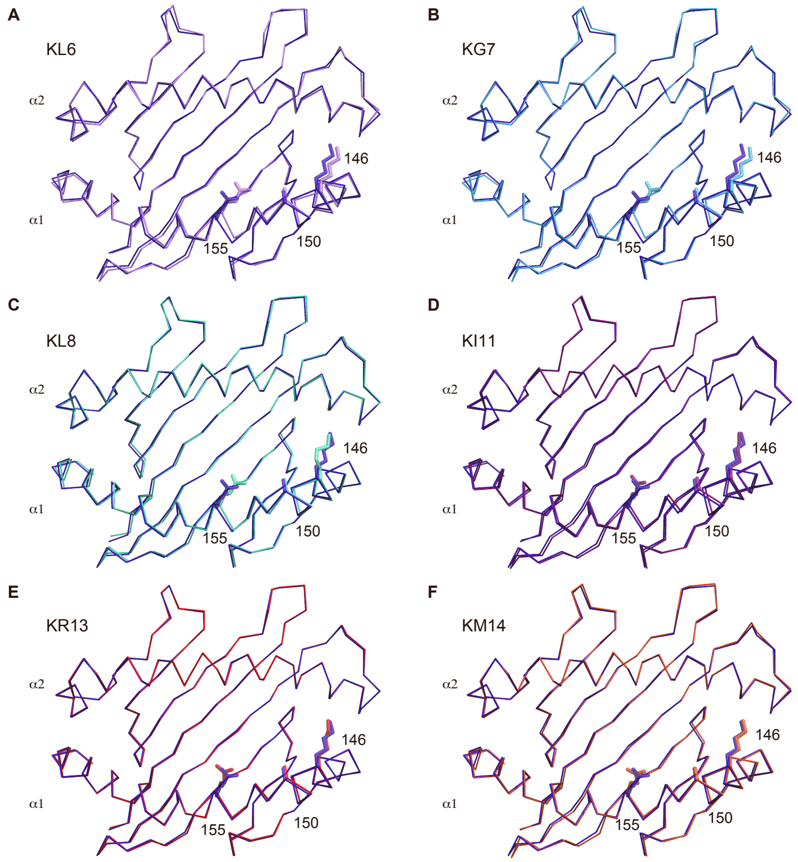

Structure colour key: KK10 = purple, KL6 = mauve, KG7 = baby blue, KL8 = turquiose, KI1 = magenta, KR13 = red, KM14 = orange

**Structure:** **Residue**	KL6	KG7	KL8	KI11	KR13	KM14
146	0.8➔	0.6➔	0.5➔	0.4↗	0.3↗	0.3↗
150	0.7➔↓	0.5➔↓	0.3➔	0.4↗	0.4↗	0.2↗
155	1.2↗	1.2↗	0.6↗	0.5↗	0.4↗	0.4↗

←	Wider
➔	Narrower
↑	Up from Platform
↓	Towards Platform
↙	N-Terminal
↗	C-Terminal

Figure S6. **The positions of the HLA helices and key residues in all structures relative to the corresponding positions in the HLA-B*27:05-KK10 structure**; (**A**), KL6 (**B**), KG7 (**C**), KL8, (**D**), KI11, (**E**), KR13, and, (**F**), KM14**, related to Figures 2, 3 and 5**. Each structure was overlaid on the α1 helix of HLA-B*27:05-KK10 (purple). Changes in the positions of key residues ($D146_{B27}$, $L150_{B27}$, and $S155_{B27}$) are summarized in the bottom panel with distances measured in Å.

Figure S7

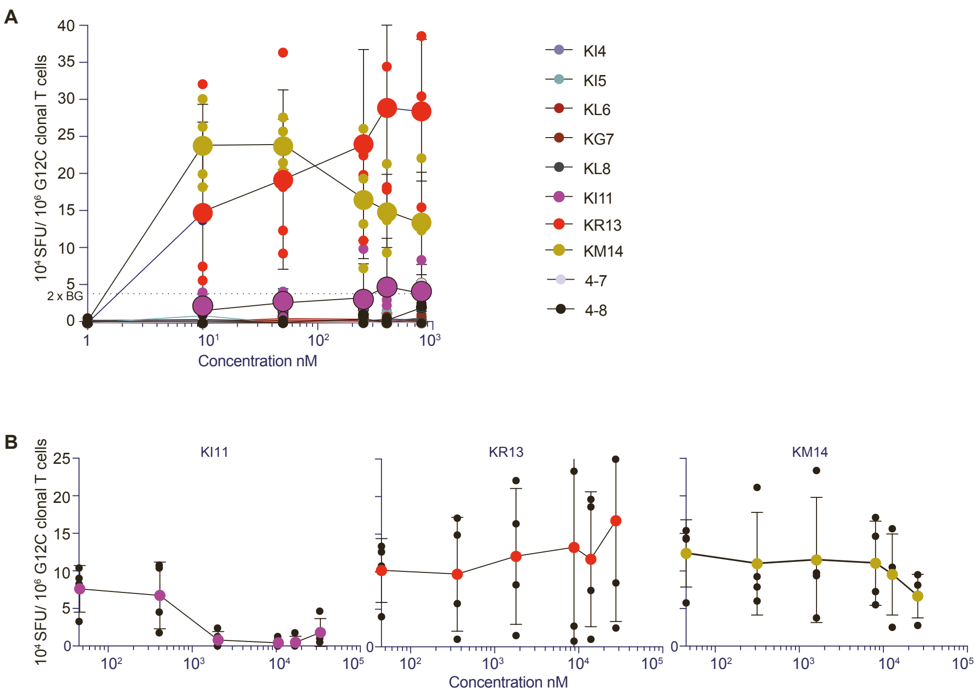

Figure S7. Analyses of G12C responses to extended KK10 epitope-form peptides. (**A**) IFN-γ release by G12C in response to individual KK10-minitopes and two mixtures of KK10-minitopes at molar ratios of 1, 10, 50, 250, 400, and 800 relative to KK10**, related to Figure 6**. The KM14 response was likely affected at higher ratios by excess DMSO (final concentration > 1%). Small dots indicate experimental data. Large dots indicate mean values. The dotted line indicates twice the background response (BG). Error bars indicate SD. (**B**) IFN-γ release by G12C in response to KK10 at the EC50 concentration in the presence of KI11, KR13, or KM14 at molar ratios of 1, 10, 50, 250, 400, and 800 relative to KK10. The KM14 response was likely affected at higher ratios by excess DMSO (final concentration > 1%). Small dots indicate experimental data. Large dots indicate mean values. Twice the background response was subtracted per data

point. Error bars indicate SD. ELISpot data are shown as spot-forming units (SFU) per 10^6 cells (A, B).

Supplementary Tables 1 to 3

Table S1. Proteasomal production of KK10 epitope-forms

(related to Fig. 1)

WT, wildtype; K, R132K substitution; M, L136M substitution; KM, R132K and L136M substitutions; CP, constitutive proteasome (data shown as % of all fragments produced); IP, immunoproteasome (data shown as % of all fragments produced). Asterisks indicate trace amounts of a given epitope-form. Red indicates the presence of a CTL escape mutation in a given fragment.

KK10 epitope-form	p24 fragment VGEIYKRWIILGLNKIVRMYSPTSI Sequence	4h CP digest %	4h IP digest %
KM-14-WT	VGEIYKRWIILGLNKIVRM	3.6	0.2
KM-14-WT (GM18)	GEIYKRWIILGLNKIVRM	0.2	0
KR-13-WT	VGEIYKRWIILGLNKIVR	2	0
KR-13-WT (GR17)	GEIYKRWIILGLNKIVR	0.1	0
KI-11-WT	VGEIYKRWIILGLNKI	1	0
KI-11-WT (GI15)	GEIYKRWIILGLNKI	0.1	0
KK-10-WT (VK15)	VGEIYKRWIILGLNK	0.2	0.1
KL-8-WT	VGEIYKRWIILGL	4.6	7.7
KL-8-WT	GEIYKRWIILGL	1.9	2.7
KL-8-WT	EIYKRWIILGL	0.5	0.8
KL-8-WT	IYKRWIILGL	0.1	0.4
KL-8-WT	YKRWIILGL	0.1	0.1
KL-8-WT	KRWIILGL	0.7	5.2
KG-7-WT	VGEIYKRWIILG	1.8	0.5
KG-7-WT	GEIYKRWIILG	1.3	0.4
KG-7-WT	KRWIILG	0.6	0.1
KL-6-WT	VGEIYKRWIIL	10	8.8
KL-6-WT	GEIYKRWIIL	10.8	9
KL-6-WT	EIYKRWIIL	1.6	1.4
KL-6-WT	IYKRWIIL	0.4	0.3
KL-6-WT	KRWIIL	3.8	5.5
KI-5-WT	VGEIYKRWII	0.2	0.5
KI-5-WT	GEIYKRWII	0.2	0.9
KI-5-WT	IYKRWII	0.1	0.2
KI-5-WT	KRWII	0.1	0.1
KI-4-WT	VGEIYKRWI	0.2	0.1
KI-4-WT	GEIYKRWI	0.3	0.1

Total KK-10 epitope-form production		46.5	45.1

KK10 epitope form	p24 fragment VGEIYKKWIILGLNKIVRMYSPTSI Sequence	4h CP digest %	4h IP digest I %
KK-10-K	VGEIYKKWIILGLNK	0.5	0.0
KL-8-K	VGEIYKKWIILGL	7.3	0.1
KL-8-K	EIYKKWIILGL	0.1	0.0
KL-8-K	KKWIILGL	0.0	0.003*
KL-6-K	VGEIYKKWIIL	24.6	0.2
KL-6-K	GEIYKKWIIL	1.4	0.006*
KL-6-K	KKWIIL	0.4	0.005*
KI-5-K	VGEIYKKWII	0.2	0.01*
KI-5-K	GEIYKKWII	0.0	0.002*
Total KK-10 epitope-form production		34.5	0.3

KK10 epitope form	p24 fragment VGEIYKRWIIMGLNKIVRMYSPTSI Sequence	4h CP digest %	4h IP digest %
KL-8-M	VGEIYKRWIIMGL	2.7	12.8
KL-8-M	KRWIIMGL	0.2	3.4
KG-7-M	VGEIYKRWIIMG	9.6	2
KG-7-M	EIYKRWIIMG	0.0	0.04*
KG-7-M	KRWIIMG	0.6	0.2
KM-6-M	VGEIYKRWIIM	11.8	6.7
KM-6-M	GEIYKRWIIM	0.4	0.3
KM-6-M	KRWIIM	0.4	4.3
KI-5-M	VGEIYKRWII	0.5	5.6
KI-5-M	GEIYKRWII	0.0	0.6
Total KK-10 epitope-form production		26.2	35.94

KK10 epitope form	p24 fragment VGEIYKKWIIMGLNKIVRMYSPTSI Sequence	4h CP digest %	4h IP digest %
KV-12-KM	GEIYKKWIIMGLNKIV	0.2	0.1
KI-11-KM	VGEIYKKWIIMGLNKI	0.2	0.1
KK-10-KM	KKWIIMGLNK	0.2	0.2
KL-8-KM	VGEIYKKWIIMGL	3.2	15.8
KL-8-KM	GEIYKKWIIMGL	0.3	0.5
KL-8-KM	KKWIIMGL	0.0	0.6
KM-6-KM	VGEIYKKWIIM	16.5	13
KM-6-KM	GEIYKKWIIM	0.7	0.5
KI-5-KM	VGEIYKKWII	1.1	8.1
KI-5-KM	GEIYKKWII	0.1	1.2
Total KK-10 epitope-form production		22.5	40.1

Table S2. Crystal data collection and refinement statistics (molecular replacement) (related to Fig. 2 and Fig. S1)

	HLA-B*27:05-KM14	HLA-B*27:05-KR13	HLA-B*27:05-KI11	HLA-B*27:05-KL8	HLA-B*27:05-KG7	HLA-B*27:05-KL6
**Data collection**						
Space group	$P2_12_12_1$	$P2_12_12_1$	$P2_12_12_1$	$P2_12_12_1$	$P2_1$	$P2_1$
**Cell dimensions**						
a, *b*, *c* (Å)	51.29, 82.81, 110.57	50.95, 82.65, 109.39	51.25, 83.07, 110.12	50.87, 82.71, 108.64	45.35, 129.86, 90.26	45.49, 130.52, 90.17
α, β, γ (°)	90, 90, 90	90, 90, 90	90, 90, 90	90, 90, 90	90, 104.38, 90	90, 104.41, 90
Resolution (Å)	66.28-2.25 (2.37-2.25)	46.18-1.77 (1.81-1.77)	66.32-2.10 (2.21-2.10)	54.32-1.88 (1.98-1.88)	87.44-2.57 (2.71-2.57)	87.33-2.25 (2.37-2.25)
R_{sym} or R_{merge}	0.44 (1.89)	0.07 (0.41)	0.21 (0.80)	0.12 (0.77)	0.23 (0.60)	0.09 (0.49)
I / σI	7.8 (3.8)	13.7 (3.9)	8.4 (2.3)	15.6 (3.6)	5.6 (2.2)	6.7 (2.3)
Completeness (%)	100 (100)	99.8 (96.7)	100 (100)	100 (100)	100 (100)	98.1 (97.1)
Redundancy	12.4 (12.2)	6.1 (4.9)	7.1 (7.2)	14.2 (14.5)	5.2 (3.5)	2.5 (2.6)
**Refinement**						
No. reflections	23098 (3309)	45852 (2489)	28236 (4054)	38122 (5467)	32272 (4694)	47317 (6830)
R_{work} / R_{free}	17.5 / 23.0	16.1 / 18.6	19.0 / 23.5	17.2 / 20.6	21.1 / 23.9	20.0 / 24.3
No. atoms	3472	3639	3522	3731	6434	6521
Protein	3198	3245	3210	3211	6305	6406
Ligand/ion	12	12	6	18	12	0
Water	262	382	306	502	117	115
B-factors	28.65	26.37	20.64	27.60	28.39	39.81
Protein	28.22	25.03	20.21	26.31	28.46	39.94
Ligand/ion	39.20	42.43	26.09	35.24	33.23	N/A
Water	33.30	37.28	25.10	35.56	24.27	32.50
R.m.s. deviations						
Bond lengths (Å)	0.004	0.008	0.014	0.009	0.003	0.004
Bond angles (°)	0.69	0.97	1.22	0.92	0.61	0.79

Table S3. Patient details (related to Fig. 5)

Patient ID	KK10 response	Years infected	Viral load copies/ml	CD4 count cells/μl	Medication
**1**	yes	9 months	112,171	330	NA
**2**	yes	11 years, 5 months	91,258	630	NA
**3**	yes	16 years, 1 month	34,704	540	NA
**4**	yes	2 years, 4 months	16,625	760	NA
**5**	yes	1 year, 5 months	12,614	540	NA
**6**	yes	4 years, 2 months	39	419	Truvada, Efavirenz
**7**	no	19 years, 7 months	39	1,000	Viread, Kaletra
**8**	no	4 years	87,342	580	NA
**9**	no	6 years	190	470	Viramun, Kivexa
**10**	no	3 to 23 years*	1,843	190	NA

Information at the time of sampling. *The first positive test was 3 years before sampling, but patient 10 was likely infected 15–20 years earlier. NA, not applicable.
